# Calcimimetic and vitamin D receptor agonist therapy associates with lower mortality and fractures in hemodialysis patients: an international DOPPS analysis

**DOI:** 10.1093/ckj/sfag164

**Published:** 2026-05-21

**Authors:** Ken Iseri, Brian Bieber, Noriko Hida

**Affiliations:** Department of Clinical Pharmacy, Division of Clinical Research and Development, School of Pharmacy, Showa Medical University, Tokyo, Japan; Jinsei-kai Kasai Dialysis Clinic, Tokyo, Japan; Arbor Research Collaborative for Health, 3989 Research Park Drive, Ann Arbor, MI 48108, USA; Department of Clinical Pharmacy, Division of Clinical Research and Development, School of Pharmacy, Showa Medical University, Tokyo, Japan

**Keywords:** calcimimetics, dialysis, fracture, mortality, VDRA

## Abstract

**Background:**

Evidence linking chronic kidney disease-mineral and bone disorder (CKD-MBD) therapies to hard clinical outcomes in hemodialysis remains limited. Treatment with vitamin D receptor activators (VDRAs) and calcimimetics is often adjusted or discontinued in response to parathyroid hormone values, which complicates the evaluation of their associations with clinical outcomes in observational research.

**Methods:**

Because VDRA/calcimimetic prescriptions change over time in response to CKD-MBD markers that also predict outcomes, data from Phases IV–VII (2009–2022) of the Dialysis Outcomes and Practice Patterns Study were analyzed using marginal structural models with time-updated, four-category exposure status (neither use, VDRA only, calcimimetics only, or both) to investigate the hazard ratios (HRs) associated with all-cause mortality, cardiovascular disease (CVD) mortality, bone fractures, and hip fractures.

**Results:**

A total of 117 452 hemodialysis patients were included in the all-cause mortality analysis, 50 111 patients in the CVD mortality analysis, and 33 906 patients in the fracture analysis. Compared with neither use, VDRA-only use (HR 0.71, *P* < .001), calcimimetics-only use (HR 0.76, *P* < .001), and combination therapy (HR 0.58, *P* < .001) were significantly associated with a reduced risk of all-cause mortality. For CVD mortality, VDRA-only (HR 0.83, *P* < .001), calcimimetics-only (HR 0.71, *P* < .001), and combination therapy (HR 0.71, *P* < .001) were significantly associated with a lower risk of CVD mortality. For bone fractures, calcimimetics-only (HR 0.59, *P* < .001) and combination therapy (HR 0.74, *P* < .01) were associated with lower risk of fracture, whereas VDRA-only was not (HR 0.89, *P* = .102). For hip fractures, only combination therapy was associated with lower hip fracture risk (HR 0.56, *P* < .01), while VDRA-only (HR 0.93, *P* = .494) and calcimimetics-only (HR 0.67, *P* = .082) were not statistically significant. Exploratory subgroup analyses by baseline characteristics showed broadly similar directions of association, although several estimates were imprecise.

**Conclusions:**

Calcimimetics and combination therapy were associated with lower fracture risk in addition to lower mortality, but these findings should be interpreted cautiously and regarded as hypothesis-generating.

## Graphical Abstract

**Graphical Abstract:**
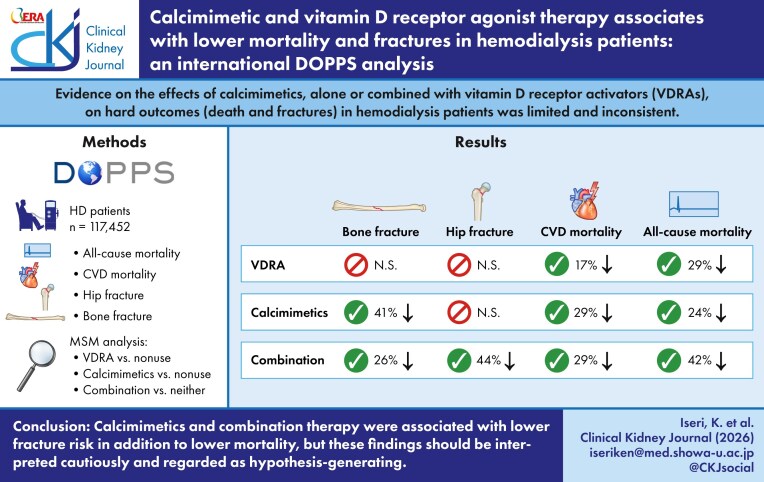

KEY LEARNING POINTS **What was known:**

- Evidence on the effects of calcimimetics, alone or combined with vitamin D receptor activators (VDRAs), on hard outcomes (death and fractures) in hemodialysis patients was limited and inconsistent.
- VDRA/calcimimetic prescriptions are frequently initiated, adjusted, or discontinued in response to evolving chronic kidney disease-mineral and bone disorder markers (especially intact parathyroid hormone), creating time-varying confounding that can bias conventional observational analyses.

**This study adds:**

- In a large international Dialysis Outcomes and Practice Patterns Study cohort analyzed using marginal structural models with time-updated treatment, VDRA-only and calcimimetic-only use were each significantly associated with lower all-cause and cardiovascular disease mortality compared with neither therapy.
- Calcimimetic-only use and combination therapy were associated with lower rates of clinically recognized fracture events, whereas VDRA-only use was not; combination therapy was additionally associated with lower hip fracture rates.

**Potential impact:**

- Calcimimetics and combination therapy were associated with lower fracture risk in addition to lower mortality in hemodialysis patients, warranting further study.

## INTRODUCTION

Chronic kidney disease-mineral and bone disorder (CKD-MBD) is a systemic condition characterized by abnormalities in mineral metabolism, bone structure, and vascular calcification in patients with end-stage kidney disease [1–3]. These disturbances include alterations in calcium, phosphorus, and parathyroid hormone (PTH) levels, which are associated with increased morbidity and mortality among dialysis patients [4]. The management of CKD-MBD remains a significant clinical challenge, with current therapeutic approaches primarily focused on vitamin D receptor activators (VDRAs) and calcimimetics [1].

VDRAs have been widely used to treat elevated intact PTH (i-PTH) levels in dialysis patients. While previous observational studies have suggested potential mortality benefits associated with VDRA use [5–7], randomized controlled trials (RCTs) did not find a significant reduction in cardiovascular disease (CVD) events or mortality [8, 9]. The J-DAVID trial, for instance, found no significant reduction in CVD events among Japanese hemodialysis patients with low PTH levels treated with VDRAs [9]. This discrepancy between observational data and clinical trials highlights the need for further investigation into the efficacy of these agents across different patient populations and i-PTH levels.

Calcimimetics are another therapeutic option for managing elevated i-PTH. Despite significant improvements in i-PTH levels observed in clinical trials [10, 11], large-scale clinical evidence regarding their impact on hard clinical outcomes remains inconsistent. The EVOLVE trial did not show a statistically significant reduction in all-cause mortality or cardiovascular events in its primary analysis, although *post hoc* analyses suggested potential benefits for clinical outcomes [12–14].

Furthermore, while both VDRAs and calcimimetics are often prescribed for CKD-MBD management, their combination has gained increasing attention in clinical practice. The complementary mechanisms of action—where VDRAs potentially increase serum calcium and phosphate while calcimimetics typically reduce them—suggest that combination therapy may theoretically optimize mineral metabolism while minimizing side effects. However, the clinical outcomes associated with this combined approach remain largely unexplored.

The present study aims to investigate the associations of VDRA use, calcimimetic use, and their combination with clinical outcomes (all-cause mortality, CVD mortality, and fracture risk) in a large international cohort of hemodialysis patients, using a time-varying marginal structural model with monthly updates to account for treatment changes driven by laboratory markers that also predict outcomes.

## MATERIALS AND METHODS

### Data source

The Dialysis Outcomes and Practice Patterns Study (DOPPS) is a prospective cohort study designed to investigate hemodialysis practices and clinical outcomes by collecting longitudinal observational data. Detailed descriptions of the study design and methodology were provided elsewhere [15]. Briefly, DOPPS collects each individual’s demographic data, comorbidities, laboratory data, medications, and prospective follow-up data for hospitalization and mortality. Since the study used de-identified data, informed consent was not required. The study protocol using DOPPS data was approved by the Showa University Ethics Committee (approval number: 2024-230-B).

### Participant selection and study design

This study used data from 131 269 hemodialysis patients enrolled in DOPPS Phases IV–VII (2009–2022). After applying the exclusion criteria, a total of 117 452 hemodialysis patients were included in the all-cause mortality analysis, 50 111 patients in the CVD mortality analysis, and 33 906 patients in the fracture analysis. A flow chart of the patient selection is shown in Supplemental Fig. S1. The CVD mortality cohort was restricted to facilities where cause of death was commonly reported, and the fracture/hip fracture cohorts to facilities where cause of hospitalization was commonly reported. Thus, the smaller CVD mortality and fracture cohorts reflect outcome availability rather than additional exclusion by the marginal structural model. The study coordinator recorded an interval summary for each sampled patient every month, along with baseline demographic information.

### Definition of exposure

At each visit, medication exposure was classified into four mutually exclusive, time-updated categories: (i) neither VDRA nor calcimimetics, (ii) VDRA only, (iii) calcimimetics only, and (iv) combination therapy (VDRA plus calcimimetics). Person-month records with missing VDRA or calcimimetic exposure data were excluded; patients could still contribute other records with observed exposure.

Treatment history was incorporated through the lagged treatment category and cumulative stabilized weights, rather than a separate duration term. “Lagged” refers to the value at the immediately preceding visit interval; for the first interval, the current value was used. Exposure was therefore characterized at the treatment-class level for each monthly interval. Exact dose, adherence, formulation-specific dose equivalence, and precise within-month start/stop dates were unavailable; thus, cumulative dose and cumulative months on therapy were not modeled directly.

### Outcomes

Our primary outcome was all-cause mortality, with secondary outcomes including CVD mortality, bone fractures, and hip fractures. CVD mortality was defined by deaths caused by stroke, myocardial infarction, hyperkalemia, hypokalemia, pericarditis, atherosclerotic heart disease, cardiomyopathy, cardiac arrhythmia, cardiac arrest, valvular heart disease, pulmonary edema from fluid overload, congestive heart failure, cerebrovascular accident, and ischemic brain damage/encephalopathy. Fracture outcomes were ascertained from emergency department or inpatient diagnosis/procedure codes recorded in DOPPS without central adjudication or imaging review. Hip fracture was defined by a diagnosis code of “hip fracture.” Similarly, any fracture was defined by “hip fracture” or “other fracture” as a diagnosis code, or with “fracture repair” as a procedure code. Kidney transplantation, transfer to another dialysis unit, or the end of the observation period for each DOPPS phase—whichever occurred first—were considered censoring events. For CVD mortality, noncardiovascular death was handled using a cause-specific hazard approach; thus, the reported estimates are cause-specific hazard ratios (HRs).

### Covariates

The following variables were collected and used as covariates: age, sex, race, country, DOPPS phase, hemodialysis vintage (years), body mass index (BMI), single-pool Kt/V, comorbidities (diabetes, hypertension, coronary heart disease, congestive heart failure, cerebrovascular disease, other CVD, peripheral vascular disease, cancer, neurologic disease, lung disease, and hip/vertebral fractures), laboratory data [hemoglobin, blood urea nitrogen (BUN), creatinine, albumin-corrected calcium, phosphate, and i-PTH] , erythropoiesis-stimulating agent (ESA) use, and phosphate binder use. These variables were updated as follows: laboratory data and medication use were basically updated monthly, Kt/V and BMI every 4 months, and comorbidities were collected only at baseline. Details of the Marginal Structural Model (MSM) weight models are described in the “Statistical analysis” section. To capture potential nonlinear relationships, lagged total calcium, phosphate, and i-PTH were modeled using natural splines (degrees of freedom (df) = 3). The spline terms were specified using the default R natural spline implementation (df = 3), with knots selected from the empirical distribution of each variable rather than prespecified clinical thresholds.

### Statistical analysis

Data were expressed as median (25th–75th percentile) for continuous variables, percentages for categorical variables, or HR, as appropriate. We summarized the distribution of the four treatment categories over follow-up using the time-updated treatment status at each visit interval.

To investigate the associations between the use of VDRA, calcimimetics, or their combination and clinical outcomes (all-cause mortality, CVD mortality, bone fractures, and hip fractures), we employed an MSM with inverse probability of treatment weighting (IPTW). The data were structured in counting process format with multiple records per patient, where each record represented a monthly interval from the patient’s baseline visit. Follow-up time was calculated as days from each patient’s first visit (baseline) to subsequent monthly visits, with the last observation extended to the date of the outcome event or censoring. This approach estimates the HR associated with drug use on clinical outcomes while accounting for time-varying confounding. MSM yields less‐biased results when used in cohorts with longitudinal data that include comprehensive information on covariates influencing treatment assignment and predicting outcomes. Observational studies have reported the usefulness of MSM as a complementary approach to RCTs, facilitating the translation and validation of trial findings in broader and more heterogeneous populations [16, 17].

For missing data, we performed multiple imputations using the chained equations (MICE) approach with 20 imputed datasets and 5 iterations to achieve convergence. Continuous variables were imputed using predictive mean matching, binary variables using logistic regression, and categorical variables using polytomous logistic regression. The imputation models included all covariates used in the subsequent analyses, as well as lagged values of time-varying covariates to preserve the longitudinal structure. The predictor matrix for MICE was specifically configured to ensure that serum calcium, phosphate, and i-PTH were robustly imputed by using them as predictors for each other, reflecting their physiological interdependencies. Exposure variables were not imputed; observations with missing exposure data were excluded from the analysis to avoid introducing additional assumptions about treatment status.

To assess the adequacy of the imputation procedure, we examined convergence using trace plots of the mean and standard deviation of imputed values across iterations for key laboratory variables and assessed distributional plausibility using density plots comparing observed and imputed values for i-PTH, calcium, phosphate, hemoglobin, and creatinine. To further evaluate the plausibility of the Missing at Random assumption for i-PTH, we compared person-month records with observed i-PTH and those with missing i-PTH before imputation and fitted a multivariable logistic regression model for i-PTH missingness. Because missingness was associated mainly with observed variables included in the imputation framework, the Missing at Random assumption was considered reasonable for the primary analysis, although it cannot be formally verified from the observed data alone. These diagnostics are shown in Supplemental Figs S2 and S3 and Supplemental Tables S1 and S2.

For the MSM analysis, we modeled treatment as a single four-category variable (neither drug, VDRA only, calcimimetics only, or both drugs) using multinomial logistic regression to estimate the treatment weights, rather than fitting separate models for each treatment comparison. This approach appropriately accounts for the mutual exclusivity of treatment categories and allows simultaneous estimation of associations across all comparisons. For MSM, we used time-varying stabilized treatment weights (IPTW) calculated at each visit interval. The numerator model included baseline demographics (age, sex, and dialysis vintage), the lagged treatment category (i.e. treatment status at the previous visit), and DOPPS phase, and country/region indicators. The denominator model included the lagged treatment category, baseline demographics, comorbidities, country, lagged values of serum calcium, phosphate, and i-PTH (modeled using natural splines with 3 degrees of freedom to capture nonlinear relationships), and other time-dependent covariates: serum hemoglobin, serum creatinine, BUN, Kt/V, BMI, phosphate binder use, and ESA use, as well as race. Stabilized weights were calculated as the ratio of the probability from the numerator model to the probability from the denominator model at each time point, and then cumulated within each patient across all observed time points to properly account for the entire treatment history. Stabilized weights were truncated at the 2nd and 98th percentiles for the time-point weights and additionally at the 1st and 99th percentiles for the cumulative stabilized weights to minimize the impact of extreme weights. Each imputed dataset was analyzed separately, and estimates were pooled using Rubin’s rules. To assess the performance of the stabilized weights, we examined absolute standardized mean differences for selected covariates included in the denominator model before weighting and after time-point and cumulative weighting in the primary all-cause mortality cohort. Because stabilized weights preserve numerator-model covariates, age, sex, and dialysis vintage were not included in this balance panel. We also summarized the distributions of the stabilized time-point and cumulative weights, including the proportion of truncated observations, in the all-cause mortality, CVD mortality, and fracture cohorts to assess practical positivity (Supplemental Fig. S9 and Supplemental Table S8).

Time-to-event outcomes were analyzed using Cox proportional hazards regression models with the time-varying MSM weights, stratified by country region to allow for region-specific baseline hazards, with robust standard errors clustered at the dialysis facility level to account for within-facility correlation. The HRs represent the association of current treatment status (e.g. currently using VDRA only versus using neither drug) with outcomes while accounting for treatment history through the cumulative time-varying weights.

For exploratory sensitivity analyses, we performed weighted Cox proportional hazard models stratified by several baseline characteristics: sex, age (<65 vs ≥65 years), BMI (<22 vs ≥22 kg/m²), baseline i-PTH (<300, 300–600, and ≥600 pg/ml), baseline phosphate (<4.5, 4.5–6.2, and >6.2 mg/dl), baseline phosphate binder use, DOPPS phase, and country group. Within each subgroup, stabilized weights were re-estimated using the same modeling framework; when applicable, the stratifying variable was excluded from the corresponding weight model. These analyses were intended to assess qualitative consistency rather than provide definitive evidence of subgroup-specific treatment effects. Furthermore, as an indication-restricted sensitivity analysis, we repeated the primary all-cause mortality MSM after restricting the cohort to patients with baseline i-PTH ≥300 pg/ml, a prespecified subgroup threshold representing patients more likely to have a biochemical indication for CKD-MBD therapy. The restriction was defined at baseline to avoid conditioning on postbaseline laboratory values that could be influenced by treatment. As a complementary sensitivity analysis for CVD mortality, we fitted a Fine–Gray subdistribution hazard model using the baseline four-category treatment definition and treating non-CVD death as the competing event. This complementary analysis was adjusted for age, sex, dialysis vintage, country/region, and DOPPS phase, and estimates were pooled across the 20 imputed datasets using Rubin’s rules. Because a Fine–Gray model with monthly time-updated internal treatment and laboratory variables is not straightforward, this analysis was intended to provide a cumulative-incidence perspective rather than replace the primary cause-specific MSM.

Statistical analyses were performed using R for Windows, version 4.4.2 (The R Foundation for Statistical Computing, Vienna, Austria). Statistical significance was set at the level of *P* < .05.

## RESULTS

### Patient characteristics

A total of 131 269 hemodialysis patients participated in DOPPS Phases IV–VII. After applying the exclusion criteria, 117 452 patients were included in the all-cause mortality analysis, 50 111 patients in the CVD mortality analysis, and 33 906 patients in the fracture analysis (Supplemental Fig. S1). Exclusion due to missing exposure data was uncommon, affecting 1.3% of person-month records and 1.6% of total potential person-months (Supplemental Table S3). Baseline characteristics for these patients are summarized in Table 1 and Supplemental Tables S4 and S5. The median age was 66.0 years [interquartile range (IQR), 54.8–75.0], and 58.4% of patients were male. The majority of patients were from the USA/Canada (74.3%). Among 117 452 patients, 57 265 used VDRA, 4234 used calcimimetics, and 14 501 used both medications at baseline. Patients who used calcimimetics only or in combination with VDRA tended to be younger, had higher BMI, serum phosphate, and i‐PTH levels, and were prescribed more phosphate binders than those who used VDRA alone or neither medication.

**Table 1:** Baseline characteristics of study patients in all-cause mortality analysis.

Characteristics		All patients	Neither use	VDRA use	Calcimimetics use	Combination use
Number		*n* = 117 452	*n* = 41 452	*n* = 57 265	*n* = 4234	*n* = 14 501
Age		66 [55, 75]	68 [57, 77]	66 [54, 75]	62 [50, 72]	62 [51, 72]
Male sex		68 586 (58.4)	24 314 (58.7)	33 734 (58.9)	2366 (55.9)	8172 (56.4)
Race (Black)		27 283 (26.3)	6364 (17.6)	14 878 (29.3)	906 (23.8)	5135 (39.0)
Country						
	USA/Canada	87 256 (74.3)	28 992 (69.9)	43 919 (76.7)	2766 (65.3)	11 579 (79.8)
	Japan/China	9971 (8.5)	3535 (8.5)	4967 (8.7)	299 (7.1)	1170 (8.1)
	Europe^a^	16 055 (13.7)	7204 (17.4)	6476 (11.3)	970 (22.9)	1405 (9.7)
	Others^b^	4170 (3.6)	1721 (4.2)	1903 (3.3)	199 (4.7)	347 (2.4)
Phase						
Phase	Ⅳ	13 683 (11.6)	5338 (12.9)	6403 (11.2)	636 (15.0)	1306 (9.0)
	V	35 260 (30.0)	12 973 (31.3)	17 682 (30.9)	1034 (24.4)	3571 (24.6)
	VI	30 667 (26.1)	10 286 (24.8)	15 204 (26.6)	1091 (25.8)	4086 (28.2)
	VII	37 842 (32.2)	12 855 (31.0)	17 976 (31.4)	1473 (34.8)	5538 (38.2)
Hemodialysis HD vintage (years)		1.86 [0.37, 4.82]	0.88 [0.26, 3.12]	1.81 [0.38, 4.45]	4.35 [1.96, 7.67]	5.17 [2.61, 8.71]
BMI (kg/m^2^)		26.38 [22.61, 31.48]	25.95 [22.30, 30.81]	26.45 [22.71, 31.57]	26.81 [22.99, 32.17]	27.24 [23.15, 32.72]
Single-pool Kt/V		1.51 [1.33, 1.70]	1.49 [1.29, 1.69]	1.51 [1.32, 1.70]	1.53 [1.35, 1.72]	1.55 [1.40, 1.73]
*Comorbidities*						
	Diabetes	64 628 (55.0)	23 230 (56.0)	32 196 (56.2)	1991 (47.0)	7211 (49.7)
	Hypertension	89 370 (76.1)	30 869 (74.5)	43 883 (76.6)	3294 (77.8)	11 324 (78.1)
	Coronary heart disease	26 510 (22.6)	9817 (23.7)	12 567 (21.9)	959 (22.6)	3167 (21.8)
	Congest heart failure	26 099 (22.2)	9804 (23.7)	12 592 (22.0)	863 (20.4)	2840 (19.6)
	Other cardiovascular disease	22 626 (19.3)	8639 (20.8)	10 330 (18.0)	926 (21.9)	2731 (18.8)
	Cerebrovascular disease	10 174 (8.7)	3939 (9.5)	4780 (8.3)	387 (9.1)	1068 (7.4)
	Peripheral vascular disease	17 350 (14.8)	6728 (16.2)	7944 (13.9)	690 (16.3)	1988 (13.7)
	Cancer	9171 (7.8)	3691 (8.9)	4140 (7.2)	338 (8.0)	1002 (6.9)
	Neurologic disease	7439 (6.3)	2888 (7.0)	3334 (5.8)	281 (6.6)	936 (6.5)
	Lung disease	9790 (8.3)	4067 (9.8)	4434 (7.7)	349 (8.2)	940 (6.5)
	History of hip/vertebral fractures	1744 (1.5)	746 (1.8)	761 (1.3)	87 (2.1)	150 (1.0)
*Laboratory*						
	Hemoglobin (g/dl)	10.70 [9.80, 11.60]	10.60 [9.60, 11.50]	10.80 [9.90, 11.60]	10.90 [9.90, 11.80]	10.80 [10.00, 11.60]
	BUN (mg/dl)	53.00 [42.00, 66.00]	52.00 [40.00, 65.33]	53.00 [42.00, 66.00]	57.00 [45.27, 70.00]	55.10 [44.00, 67.67]
	Cre (mg/dl)	7.39 [5.50, 9.60]	6.42 [4.74, 8.60]	7.48 [5.70, 9.60]	8.74 [6.86, 10.82]	9.10 [7.30, 11.27]
	Corrected calcium (mg/dl)	8.90 [8.44, 9.40]	8.90 [8.40, 9.30]	8.96 [8.50, 9.40]	8.90 [8.30, 9.40]	8.92 [8.40, 9.48]
	Phosphorus (mg/dl)	5.00 [4.10, 6.10]	4.77 [3.90, 5.80]	5.00 [4.20, 6.10]	5.40 [4.30, 6.80]	5.30 [4.40, 6.40]
	i-PTH (pg/ml)	295 [167, 501]	203 [113, 335]	333 [202, 519]	469 [246, 945]	469 [255, 827]
*Medications*						
	ESA use	100 751 (86.7)	35 046 (85.6)	49 902 (88.0)	3425 (81.7)	12 378 (86.2)
	Phosphate-binder use	80 293 (68.5)	24 110 (58.4)	40 258 (70.4)	3475 (82.4)	12 450 (86.0)

Values are median [IQR], or number (proportion).

a Europe: Belgium, France, Germany, Italy, Spain, Sweden, and UK.

b Others: Australia, New Zealand, GCC, Russia, and Turkey.

Ca, albumin-corrected serum calcium; Cre, serum creatinine; HD vintage, years since initiation of maintenance haemodialysis; spKt/V, single-pool Kt/V (dialysis adequacy index); SMD, standardized mean difference.

**Figure 1:**
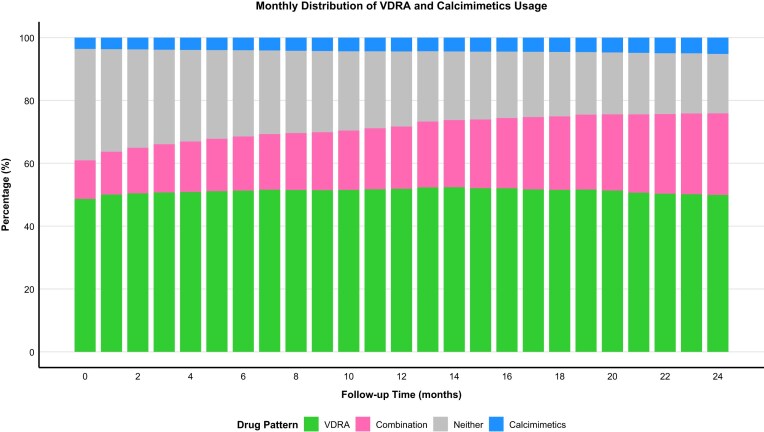
Patterns of VDRA and/or calcimimetics use during 24-month follow-up. To evaluate temporal trends in drug utilization, we analyzed the monthly distribution of VDRA and calcimimetic usage over a 24-month follow-up period. Patients were categorized into four groups based on their medication status at each visit: VDRA only, calcimimetics only, both drugs (combination therapy), neither drug. Follow-up time was calculated from each patient’s baseline visit to subsequent visits. The 100% stacked bars depict, for each monthly visit (*x*-axis, 0–24 months), the proportion of patients who were receiving combination therapy (concurrent VDRA + calcimimetic; pink), VDRA only (VDRA without calcimimetic; green), calcimimetic only (calcimimetic without VDRA; blue), or neither agent (gray). Each bar therefore sums to 100% on the *y*-axis (percentage of the cohort).

**Figure 2:**
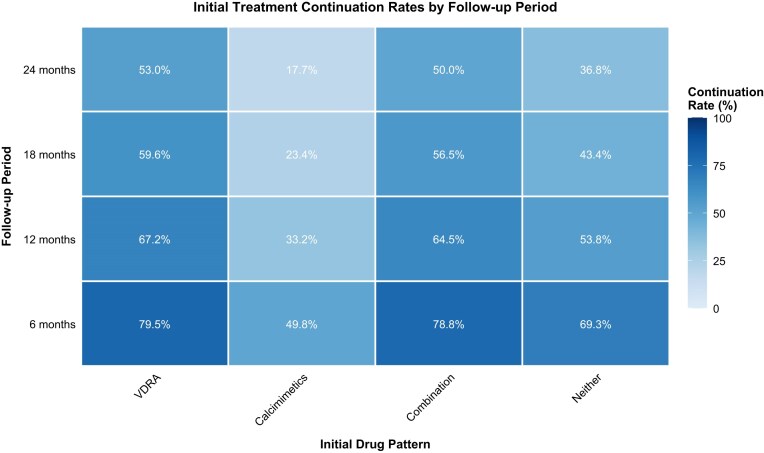
Initial treatment continuation rates by follow-up period. To evaluate the persistence of initial treatment strategies, we analyzed the proportion of patients who maintained their baseline drug pattern throughout specified follow-up periods (6, 12, 18, and 24 months). For patients with both medications missing at a given visit, we applied last observation carried forward imputation to minimize data loss (only for this analysis). Continuation rates were calculated as the percentage of patients with no change in drug pattern from baseline to each time point, restricted to patients with at least 80% of the specified follow-up duration. The heat-map shows the percentage of patients who continued the same regimen they used at baseline (columns) at 6, 12, 18, and 24 months of follow-up (rows). Cell shading follows a sequential blue scale (legend at right): white ≈0% and navy ≈100% continuation. Exact continuation rates are printed inside each cell. Patients with <80% of the respective follow-up period were excluded from each time-point analysis to ensure reliable estimation of continuation rates. Grid lines are plain white; no additional line styles or symbols are used.

Time courses of the CKD-MBD parameters (serum i-PTH levels, calcium levels, and phosphate levels) are shown in Supplemental Fig. S4, which indicates these parameters remained stable within the recommended guideline range. As for trends in drug-usage patterns over the study period (Fig. 1 and Supplemental Table S6), the percentage of patients using VDRA only remained stable. Conversely, the percentage of patients receiving combination therapy gradually increased, while the proportion of neither medication decreased over time. Furthermore, we examined the persistence of initial treatment strategies over the study period. Overall, 70 985 of 117 452 patients (60.4%) had no change in their initial drug pattern during observed follow-up. Regimen-specific continuation over 6, 12, 18, and 24 months is shown in Fig. 2 and Supplemental Table S7. By usage pattern, VDRA only showed the highest continuation rate of 63.9% (36 601 of 57 265 patients), followed by neither use at 59.1% (24 494 of 41 452 patients), then the combination group at 57.4% (8320 of 14 501 patients). Calcimimetics only showed the lowest continuation rate of 37.1%; only 1570 of 4234 continued. Supplementary diagnostics showed that the stabilized weights were generally centered near 1 across outcome cohorts after truncation, although truncation was more frequent for calcimimetic-only observations. Balance improved for several selected denominator-model covariates after weighting but remained incomplete for comparisons involving calcimimetic-containing regimens, particularly for serum creatinine and lagged i-PTH (Supplemental Fig. S9 and Supplemental Table S8).

### Primary outcome

#### All-cause mortality

In the weighted analyses, the all-cause mortality rate was highest among patients receiving neither VDRA nor calcimimetics [18.39 per 100 patient-years, 95% confidence interval (CI) 17.95–18.84] and lowest among those receiving combination therapy (11.56 per 100 patient-years, 95% CI 11.17–11.97) (Table 2). Compared with neither use, VDRA-only use was associated with a significantly lower risk of all-cause mortality (HR 0.71, 95% CI 0.68–0.74; *P* < .001). Calcimimetics-only use was also associated with lower all-cause mortality (HR 0.76, 95% CI 0.70–0.82; *P* < .001). The strongest association was observed for combination therapy (HR 0.58, 95% CI 0.55–0.61; *P* < .001) (Table 2). In exploratory subgroup analyses based on baseline characteristics (sex, age, BMI, i-PTH, DOPPS phase, and country region), the associations of VDRA-only use and combination therapy with all-cause mortality were generally consistent across strata, whereas estimates for calcimimetics-only use were less precise and not consistently statistically significant in some strata (Supplemental Fig. S5). In an indication-restricted sensitivity analysis limited to patients with baseline i-PTH ≥300 pg/ml [57 425/117 452 patients (48.9%)], the associations with all-cause mortality were attenuated but remained directionally similar: HR 0.75 (95% CI 0.70–0.80) for VDRA-only use, 0.81 (95% CI 0.73–0.90) for calcimimetics-only use, and 0.62 (95% CI 0.58–0.67) for combination therapy versus neither use (Supplemental Table S9).

**Table 2:** Association of VDRA and calcimimetic use with all-cause mortality.

Exposure	Number of patients^a^, *n*	Number of events^a^, *n*	Follow-up mean (SD)^a^, months	Event rate^a^, 100 patient-years (95% CI)	HR (95% CI)			
					Crude^b^	*P*-value	Weighted^c^	*P*-value
Neither use	54 412	6636	8.0 (8.5)	18.39 (17.95–18.84)	ref		ref	
VDRA only	73 596	10 023	11.9 (10.7)	13.75 (13.48–14.02)	0.67 (0.64–0.69)	<.001	0.71 (0.68–0.74)	<.001
Calcimimetics only	11 202	870	6.4 (7.1)	14.58 (13.63–15.58)	0.75 (0.69–0.81)	<.001	0.76 (0.70–0.82)	<.001
Combination therapy	25 709	3219	13.0 (11.2)	11.56 (11.17–11.97)	0.53 (0.51–0.56)	<.001	0.58 (0.55–0.61)	<.001

^a^Number of patients, events, follow-up periods, and event rate were calculated in the weighted population.

^b^Crude models were stratified by country region and used robust variance estimation with clustering at the facility level.

^c^Adjustments for confounders: age, sex, race, country, DOPPS phase, hemodialysis vintage (years), BMI, single-pool Kt/V, comorbidities (diabetes, hypertension, coronary heart disease, congestive heart failure, cerebrovascular disease, other cardiovascular disease, peripheral vascular disease, cancer, neurologic disease, lung disease, and hip/vertebral fractures), laboratory data (hemoglobin, BUN, creatinine, albumin-corrected calcium, phosphate, and i-PTH ), ESA use, and phosphate binder use. The weighted Cox models were stratified by country region and used robust variance estimation with clustering at the facility level.

SD, standard deviation; ref, reference group.

### Secondary outcome

#### CVD mortality

For cardiovascular mortality, the weighted event rate was 5.25 per 100 patient-years (95% CI 4.93–5.59) in the neither-use group, compared with 4.59 (95% CI 4.37–4.82), 4.09 (95% CI 3.41–4.86), and 4.10 (95% CI 3.76–4.46) per 100 patient-years in the VDRA-only, calcimimetics-only, and combination-therapy groups, respectively (Table 3). Compared to neither use, VDRA-only use was significantly associated with lower CVD mortality (HR 0.83, 95% CI 0.76–0.91; *P* < .001). Calcimimetics-only use was also associated with lower CVD mortality (HR 0.71, 95% CI 0.59–0.86; *P* < .001), as was combination therapy (HR 0.71, 95% CI 0.63–0.79; *P* < .001) (Table 3). Exploratory subgroup analyses showed generally similar directions of association; however, several subgroup estimates were imprecise and statistical significance was not consistent across comparisons (Supplemental Fig. S6). A complementary Fine–Gray sensitivity analysis based on baseline treatment category, treating non-CVD death as the competing event, yielded attenuated subdistribution HRs versus neither use: 0.94 (95% CI 0.87–1.03) for VDRA only, 0.99 (95% CI 0.81–1.22) for calcimimetics only, and 0.94 (95% CI 0.83–1.07) for combination therapy (Supplemental Fig. S10 and Supplemental Table S10).

**Table 3:** Association of VDRA and calcimimetic use with CVD mortality.

Exposure	Number of patients^a^, *n*	Number of events^a^, *n*	Follow-up mean (SD)^a^, months	Event rate^a^, 100 patient-years (95% CI)	HR (95% CI)			
					Crude^b^	*P*-value	Weighted^c^	*P*-value
Neither use	24 229	1008	9.5 (9.2)	5.25 (4.93–5.59)	ref		ref	
VDRA only	31 979	1587	13.0 (10.9)	4.59 (4.37–4.82)	0.79 (0.72–0.87)	<.001	0.83 (0.76–0.91)	<.001
Calcimimetics only	4939	128	7.6 (7.7)	4.09 (3.41–4.86)	0.71 (0.58–0.87)	<.01	0.71 (0.59–0.86)	<.001
Combination therapy	11 166	537	14.1 (11.4)	4.10 (3.76–4.46)	0.66 (0.59–0.75)	<.001	0.71 (0.63–0.79)	<.001

^a^Number of patients, events, follow-up periods, and event rate were calculated in the weighted population.

^b^Crude models were stratified by country region and used robust variance estimation with clustering at the facility level.

^c^Adjustments for confounders: age, sex, race, country, DOPPS phase, hemodialysis vintage (years), BMI, single-pool Kt/V, comorbidities (diabetes, hypertension, coronary heart disease, congestive heart failure, cerebrovascular disease, other cardiovascular disease, peripheral vascular disease, cancer, neurologic disease, lung disease, and hip/vertebral fractures), laboratory data (hemoglobin, BUN, creatinine, albumin-corrected calcium, phosphate, and i-PTH ), ESA use, and phosphate binder use. The weighted Cox models were stratified by country region and used robust variance estimation with clustering at the facility level.

SD, standard deviation; ref, reference group.

#### Bone fractures

The event rate for any bone fracture was 2.88 per 100 patient-years (95% CI 2.62–3.16) in the neither-use group (Table 4). VDRA-only use was not significantly associated with fracture risk compared with neither use (HR 0.89, 95% CI 0.78–1.02; *P* = .102). In contrast, calcimimetics-only use and combination therapy were significantly associated with a lower fracture risk (calcimimetics-only: HR 0.59, 95% CI 0.43–0.80; *P* < .001; combination therapy: HR 0.74, 95% CI 0.60–0.91; *P* < .01) (Table 4). Exploratory subgroup analyses for fracture outcomes showed wide CIs and no consistent pattern across strata; therefore, these results should be interpreted cautiously (Supplemental Fig. S7). <?vsp -6pt?>

**Table 4:** Association of VDRA and calcimimetic use with bone fractures.

Exposure	Number of patients^a^, *n*	Number of events^a^, *n*	Follow-up mean (SD)^a^, months	Event rate^a^, 100 patient-years (95% CI)	HR (95% CI)			
					Crude^b^	*P*-value	Weighted^c^	*P*-value
Neither use	18 690	450	10.0 (9.1)	2.88 (2.62–3.16)	ref		ref	
VDRA only	20 443	520	12.5 (10.0)	2.45 (2.24–2.67)	0.89 (0.77–1.02)	.093	0.89 (0.78–1.02)	.102
Calcimimetics only	3714	46	8.4 (8.0)	1.76 (1.29–2.35)	0.59 (0.43–0.81)	<.01	0.59 (0.43–0.80)	<.001
Combination therapy	6209	133	13.1 (10.3)	1.96 (1.64–2.32)	0.73 (0.59–0.91)	<.01	0.74 (0.60–0.91)	<.01

^a^Number of patients, events, follow-up periods, and event rate were calculated in the weighted population.

^b^Crude models were stratified by country region and used robust variance estimation with clustering at the facility level.

^c^Adjustments for confounders: age, sex, race, country, DOPPS phase, hemodialysis vintage (years), BMI, single-pool Kt/V, comorbidities (diabetes, hypertension, coronary heart disease, congestive heart failure, cerebrovascular disease, other cardiovascular disease, peripheral vascular disease, cancer, neurologic disease, lung disease, and hip/vertebral fractures), laboratory data (hemoglobin, BUN, creatinine, albumin-corrected calcium, phosphate, and i-PTH ), ESA use, and phosphate binder use. The weighted Cox models were stratified by country region and used robust variance estimation with clustering at the facility level.

SD, standard deviation; ref, reference group.

#### Hip fracture

For hip fractures, event rates were 1.10 per 100 patient-years (95% CI 0.94–1.27) in the neither-use group, and lowest in the combination therapy group (0.55 per 100 patient-years, 95% CI 0.39–0.75) (Table 5). VDRA-only use was not significantly associated with hip fracture risk (HR 0.93, 95% CI 0.75–1.15; *P* = .494). Calcimimetics-only use showed a nonsignificant trend toward lower hip fracture risk (HR 0.67, 95% CI 0.43–1.05; *P* = .082). Combination therapy was significantly associated with lower hip fracture risk (HR 0.56, 95% CI 0.40–0.79; *P* < .01). Exploratory subgroup analyses for hip fracture were especially imprecise because of limited events and should be interpreted cautiously (Supplemental Fig. S8).

**Table 5:** Association of VDRA and calcimimetic use with hip fractures.

Exposure	Number of patients^a^, *n*	Number of events^a^, *n*	Follow-up mean (SD)^a^, months	Event rate^a^, 100 patient-years (95% CI)	HR (95% CI)			
					Crude^b^	*P*-value	Weighted^c^	*P*-value
Neither use	18 749	174	10.1 (9.2)	1.10 (0.94–1.27)	ref		ref	
VDRA only	20 501	206	12.6 (10.0)	0.96 (0.83–1.10)	0.90 (0.72–1.13)	.339	0.93 (0.75–1.15)	.494
Calcimimetics only	3742	20	8.4 (8.0)	0.77 (0.47–1.19)	0.63 (0.39–1.01)	.057	0.67 (0.43–1.05)	.082
Combination therapy	6235	38	13.2 (10.3)	0.55 (0.39–0.75)	0.54 (0.37–0.77)	<.01	0.56 (0.40–0.79)	<.01

^a^Number of patients, events, follow-up periods, and event rate were calculated in the weighted population.

^b^Crude models were stratified by country region and used robust variance estimation with clustering at the facility level.

^c^Adjustments for confounders: age, sex, race, country, DOPPS phase, hemodialysis vintage (years), BMI, single-pool Kt/V, comorbidities (diabetes, hypertension, coronary heart disease, congestive heart failure, cerebrovascular disease, other cardiovascular disease, peripheral vascular disease, cancer, neurologic disease, lung disease, and hip/vertebral fractures), laboratory data (hemoglobin, BUN, creatinine, albumin-corrected calcium, phosphate, and i-PTH ), ESA use, and phosphate binder use. The weighted Cox models were stratified by country region and used robust variance estimation with clustering at the facility level.

SD, standard deviation; ref, reference group.

## DISCUSSION

This study, using DOPPS data, found that VDRA use was significantly associated with lower hazards of all-cause and CVD mortality, but not with lower fracture risk. Calcimimetics use was associated with lower hazards of all-cause mortality, CVD mortality, and bone fractures. Combination therapy was associated with lower hazards of all-cause mortality, CVD mortality, bone fractures, and hip fractures; however, these associations, particularly for all-cause mortality, should be interpreted cautiously because residual confounding cannot be excluded.

Guidelines differ slightly in their recommended ranges for calcium, phosphate, and i-PTH, but there is no major difference in the treatment strategy [1, 18]. For all-cause mortality against VDRA, large observational studies [Fresenius, DARWIN, and the Japanese Society for Dialysis Therapy (JSDT)] reported that VDRA use was associated with lower all-cause mortality [5–7]. A meta-analysis further confirmed the survival benefit of VDRA use [19]. However, a randomized trial in 299 hemodialysis patients with vitamin D insufficiency was underpowered to confirm mortality or CVD benefit [8]. The benefit of VDRA on CVD mortality therefore remains controversial. Large-scale observational studies have suggested beneficial effects; however, J-DAVID RCT showed no significant association between VDRA use and decreased MACE risk [6, 9, 20]. Furthermore, observational studies using JSDT data and DOPPS Phase I–III data analyses showed no significant association with reduced CVD mortality risk, suggesting that unmeasured confounding may contribute to the apparent benefit observed in some studies. Our MSM analysis using time-updated CKD-MBD markers suggested a favorable association between VDRA use and CVD mortality. Taken together with prior null trials, these observational findings are consistent with the absence of harm but cannot establish benefit, given residual confounding. Exploratory subgroup analyses suggested broadly similar directions of association across many baseline strata, but several subgroup estimates were imprecise and not consistently statistically significant. Therefore, these analyses should be regarded as hypothesis-generating rather than as evidence of true subgroup differences. Regarding bone fracture outcomes, previous studies using DOPPS Phases III–VI data and a meta-analysis found no significant association [21, 22], which was supported by our findings. Furthermore, our finding that calcimimetics use and combination therapy, rather than VDRAs alone, were associated with a lower risk of fractures is remarkable.

Cinacalcet was associated with a reduced risk of all-cause mortality in a large observational study from Sweden (*n* = 3526) and a lower risk of MACE in a large observational study from the USA (*n* = 19 186) [23, 24]. In contrast, the EVOLVE trial (*n* = 3883) found no significant difference for all-cause mortality or MACE; however, high discontinuation in the cinacalcet arm (62%), commercial cinacalcet use in the placebo arm (19.8%), and the intention-to-treat approach may have contributed to these null findings. In our analysis, calcimimetic-only use showed the lowest continuation rate among the treated regimens, consistent with previous reports of high discontinuation and reinitiation of cinacalcet [25]. Because calcimimetics are frequently discontinued or supplemented with VDRA in response to hypocalcemia or insufficient PTH control, a time-dependent exposure definition may be more appropriate than an intention-to-treat approach to minimize misclassification. In this framework, calcimimetics use was associated with a reduced all-cause mortality risk and CVD mortality risk. For fracture outcomes, *post hoc* analyses of the EVOLVE trial showed cinacalcet-reduced fracture risk after baseline adjustment, whereas the intention-to-treat analysis did not show a significant difference [13]. Mechanistically, higher i-PTH has been associated with negative changes in cortical and trabecular bone compartments and estimated bone-strength indices at the hip region in dialysis patients [26]. In addition, calcimimetics reduce bone turnover markers and improve bone mineral density and trabecular quality [10, 27]. Lower i-PTH has also been associated with lower fracture risk in DOPPS and JSDT data. These findings support the rationale that calcimimetic-containing regimens may reduce fracture risk by decreasing i-PTH. Thus, calcimimetic-containing regimens, but not VDRA alone, were associated with lower hazards of clinically recognized fracture events. Because events were defined from diagnosis/procedure codes without imaging adjudication, these associations are hypothesis-generating rather than direct evidence of improved bone health.

Combination therapy could possibly minimize calcium/phosphate fluctuations, as VDRA and calcimimetics have distinct effects on serum calcium and phosphate levels. Moreover, an RCT showed that combination therapy had significantly higher rates of achieving an optimal i‐PTH range compared to the control group (standard therapy using VDRA) [28]. However, evidence regarding the association of combination therapy with clinical outcomes is sparse. In our study, combination therapy was associated with lower hazards of all-cause mortality, CVD mortality, and fracture outcomes, but the all-cause mortality estimate (HR 0.58) was substantial for an observational analysis and should be interpreted cautiously. MSM can address measured time-varying confounders, but patients selected for combination therapy may still have differed from those receiving neither drug in ways not fully captured in DOPPS, including frailty, nutritional status, and overall care intensity. Thus, even after weighting, part of the observed association may reflect residual confounding and care-related selection rather than a direct drug effect, and the true effect, if any, may be smaller than the observed estimate. These findings should therefore be viewed as hypothesis-generating. Another interpretive challenge is that the “neither use” group likely included patients with lower biochemical indication for CKD-MBD therapy and therefore should not be interpreted as a clinically matched untreated comparator. In a sensitivity analysis restricted to patients with baseline i-PTH ≥300 pg/ml, the mortality associations were attenuated but remained directionally similar, suggesting that this issue may explain part, but not all, of the observed associations. Residual confounding and limited overlap may still remain. Future studies using target trial emulation or analyses restricted to patients with comparable treatment indications may help clarify whether this large mortality association persists.

The strengths of the present study include the use of one of the largest prospective cohort studies focusing on hemodialysis patients worldwide. In addition, DOPPS is a rich source of data including Kt/V, laboratory values, comorbidities, and BMI, which are known to be associated with clinical outcomes. A major strength is the monthly time-varying four-category MSM framework comprising multinomial treatment models, stabilized IPTW, and weighted Cox models, which reflects routine CKD-MBD care and addresses confounding from laboratory markers that both guide treatment and predict outcomes.

Some limitations should be discussed when interpreting the results of the present study. First, as in any observational study, we cannot conclude on causality. In addition, patients receiving VDRAs and/or calcimimetics may have undergone closer CKD-MBD monitoring, more frequent treatment adjustment, dietary counseling, or better overall nephrology follow-up than untreated patients. Although we adjusted for many measured baseline and time-updated clinical factors, unmeasured differences in care intensity, care quality, or patient engagement may still have contributed to the observed associations. Second, supplementary MSM diagnostics suggested limited overlap and incomplete covariate balance for calcimimetic-containing regimens versus neither treatment; therefore, residual confounding may remain despite weighting, particularly for the large all-cause mortality association observed with combination therapy. Third, exposure characterization was limited to class-level monthly treatment status. Exact dose, adherence, cumulative dose, and cumulative months on therapy were unavailable, and short interruptions occurring entirely within a month could not be captured. Therefore, some exposure misclassification may remain. Fourth, fracture outcomes were available only at facilities reporting cause of hospitalization and were defined from diagnosis/procedure codes. We could not distinguish traumatic from fragility fractures, validate events against imaging, or capture asymptomatic vertebral fractures. Even hip fracture cannot be assumed to reflect CKD-MBD-related fragility alone, and generalizability to the full DOPPS cohort may be limited. Fifth, subgroup analyses were exploratory, and some strata—especially for fracture outcomes—had limited numbers of events; therefore, subgroup-specific differences should not be overinterpreted. Finally, although we additionally performed a complementary Fine–Gray analysis based on baseline treatment category to present the cumulative-incidence perspective, this analysis addressed a different estimand and did not preserve the full monthly time-updated MSM structure. Therefore, the primary inference for CVD mortality remains based on the cause-specific MSM.

In conclusion, time-updated use of VDRA, calcimimetics, and especially combination therapy was associated with lower hazards of all-cause and CVD mortality; calcimimetic-containing regimens were additionally associated with lower fracture-related event rates. However, the magnitude of these associations, particularly for combination therapy, should be interpreted cautiously because residual confounding by care intensity and other unmeasured factors cannot be excluded. These findings are hypothesis-generating and warrant further evaluation, ideally using target trial emulation or indication-restricted analyses.

## Supplementary Material

sfag164_Supplemental_File

## Data Availability

Restrictions apply to the availability of the data analyzed in this study to preserve patient confidentiality. Data will be shared on request to the corresponding author with permission from the DOPPS investigators.

## References

[1] Ketteler M, Evenepoel P, Holden RM et al. Chronic kidney disease-mineral and bone disorder: conclusions from a Kidney Disease: Improving Global Outcomes (KDIGO) Controversies Conference. Kidney Int. 2025;107:405–23. 10.1016/j.kint.2024.11.013 39864017

[2] Mazzaferro S, Tartaglione L, Cohen-Solal M et al. Pathophysiology and therapies of CKD-associated secondary hyperparathyroidism. Clin Kidney J. 2025;18:i15–26. 10.1093/ckj/sfae423 40083954 PMC11903092

[3] Cannata-Andía JB, Martín-Carro B, Martín-Vírgala J et al. Chronic kidney disease-mineral and bone disorders: pathogenesis and management. Calcif Tissue Int. 2021;108:410–22. 10.1007/s00223-020-00777-1 33190187

[4] Tentori F, Blayney MJ, Albert JM et al. Mortality risk for dialysis patients with different levels of serum calcium, phosphorus, and PTH: the Dialysis Outcomes and Practice Patterns Study (DOPPS). Am J Kidney Dis. 2008;52:519–30. 10.1053/j.ajkd.2008.03.020 18514987

[5] Obi Y, Hamano T, Wada A et al. Vitamin D receptor activator use and cause-specific death among dialysis patients: a nationwide cohort study using coarsened exact matching. Sci Rep. 2017;7:41170. 10.1038/srep41170 28139665 PMC5282519

[6] Teng M, Wolf M, Ofsthun MN et al. Activated injectable vitamin D and hemodialysis survival: a historical cohort study. J Am Soc Nephrol. 2005;16:1115–25. 10.1681/ASN.2004070573 15728786

[7] Tentori F, Hunt WC, Stidley CA et al. Mortality risk among hemodialysis patients receiving different vitamin D analogs. Kidney Int. 2006;70:1858–65. 10.1038/sj.ki.5001868 17021609

[8] Morrone L, Palmer SC, Saglimbene VM et al. Calcifediol supplementation in adults on hemodialysis: a randomized controlled trial. J Nephrol. 2022;35:517–25. 10.1007/s40620-021-01104-z 34173940

[9] J-DAVID Investigators, Shoji T, Inaba M et al. Effect of oral alfacalcidol on clinical Outcomes in patients without secondary hyperparathyroidism receiving maintenance hemodialysis: the J-DAVID randomized clinical trial. JAMA. 2018;320:2325–34. 10.1001/jama.2018.17749 30535217 PMC6583075

[10] Shigematsu T, Koiwa F, Isaka Y et al. Efficacy and safety of upacicalcet in hemodialysis patients with secondary hyperparathyroidism: a randomized placebo-controlled trial. Clin J Am Soc Nephrol. 2023;18:1300–9. 10.2215/CJN.0000000000000253 37696667 PMC10578632

[11] Block GA, Bushinsky DA, Cheng S et al. Effect of etelcalcetide vs cinacalcet on serum parathyroid hormone in patients receiving hemodialysis with secondary hyperparathyroidism: a randomized clinical trial. JAMA. 2017;317:156–64. 10.1001/jama.2016.19468 28097356

[12] EVOLVE Trial Investigators, Chertow GM, Block GA et al. Effect of cinacalcet on cardiovascular disease in patients undergoing dialysis. N Engl J Med. 2012;367:2482–94. 10.1056/NEJMoa1205624 23121374

[13] Moe SM, Abdalla S, Chertow GM et al. Effects of cinacalcet on fracture events in patients receiving hemodialysis: the EVOLVE trial. J Am Soc Nephrol. 2015;26:1466–75. 10.1681/ASN.2014040414 25505257 PMC4446874

[14] Parfrey PS, Drüeke TB, Block GA et al. The effects of cinacalcet in older and younger patients on hemodialysis: the evaluation of cinacalcet HCl therapy to lower cardiovascular events (EVOLVE) trial. Clin J Am Soc Nephrol. 2015;10:791–9. 10.2215/CJN.07730814 25710802 PMC4422239

[15] Young EW, Goodkin DA, Mapes DL et al. The Dialysis Outcomes and Practice Patterns Study (DOPPS): an international hemodialysis study. Kidney Int. 2000;57:S74–81. 10.1046/j.1523-1755.2000.07413.x

[16] Cole SR, Hernán MA, Anastos K et al. Determining the effect of highly active antiretroviral therapy on changes in human immunodeficiency virus type 1 RNA viral load using a marginal structural left-censored mean model. Am J Epidemiol. 2007;166:219–27. 10.1093/aje/kwm047 17478436

[17] Cole SR, Hudgens MG, Tien PC et al. Marginal structural models for case-cohort study designs to estimate the association of antiretroviral therapy initiation with incident AIDS or death. Am J Epidemiol. 2012;175:381–90. 10.1093/aje/kwr346 22302074 PMC3282878

[18] Fukagawa M, Yokoyama K, Koiwa F et al. Clinical practice guideline for the management of chronic kidney disease-mineral and bone disorder. Ther Apher Dial. 2013;17:247–88. 10.1111/1744-9987.12058 23735142

[19] Duranton F, Rodriguez-Ortiz ME, Duny Y et al. Vitamin D treatment and mortality in chronic kidney disease: a systematic review and meta-analysis. Am J Nephrol. 2013;37:239–48. 10.1159/000346846 23467111

[20] Naves-Díaz M, Alvarez-Hernández D, Passlick-Deetjen J et al. Oral active vitamin D is associated with improved survival in hemodialysis patients. Kidney Int. 2008;74:1070–8. 10.1038/ki.2008.343 18633342

[21] Komaba H, Zhao J, Karaboyas A et al. Active vitamin D use and fractures in hemodialysis patients: results from the international DOPPS. J Bone Miner Res. 2023;38:1577–85. 10.1002/jbmr.4913 37718534

[22] Khelifi N, Desbiens LC, Sidibé A et al. Vitamin D analogues and fracture risk in chronic kidney disease: a systematic review and meta-analysis of randomized controlled trials. JBMR Plus. 2022;6:e10611. 10.1002/jbm4.10611 35434454 PMC9009117

[23] Evans M, Methven S, Gasparini A et al. Cinacalcet use and the risk of cardiovascular events, fractures and mortality in chronic kidney disease patients with secondary hyperparathyroidism. Sci Rep. 2018;8:2103. 10.1038/s41598-018-20552-5 29391567 PMC5794851

[24] Block GA, Zaun D, Smits G et al. Cinacalcet hydrochloride treatment significantly improves all-cause and cardiovascular survival in a large cohort of hemodialysis patients. Kidney Int. 2010;78:578–89. 10.1038/ki.2010.167 20555319

[25] Fuller DS, Hallett D, Dluzniewski PJ et al. Predictors of cinacalcet discontinuation and reinitiation in hemodialysis patients: results from 7 European countries. BMC Nephrol. 2019;20:169. 10.1186/s12882-019-1355-5 31088377 PMC6518810

[26] Iseri K, Mizobuchi M, Shishido K et al. Association between CKD-MBD and hip-bone microstructures in dialysis patients. Clin Kidney J. 2024;17:sfae240. 10.1093/ckj/sfae240 39188768 PMC11345638

[27] Khairallah P, Cherasard J, Sung J et al. Changes in bone quality after treatment with etelcalcetide. Clin J Am Soc Nephrol. 2023;18:1456–65. 10.2215/CJN.0000000000000254 37574661 PMC10637456

[28] Itano Y, Kato S, Tsuboi M et al. A prospective, randomized clinical trial of etelcalcetide in patients receiving hemodialysis with secondary hyperparathyroidism (the DUET trial). Kidney Int Rep. 2020;5:2168–77. 10.1016/j.ekir.2020.09.010 33305109 PMC7710846

