# Supplementary material for: Calcimimetic and vitamin D receptor agonist therapy associates with lower mortality and fractures in hemodialysis patients: an international DOPPS analysis
Source: Clin Kidney J. 2026 May 21;19(7):sfag164. doi: 10.1093/ckj/sfag164 (PMC13329188; doi:10.1093/ckj/sfag164)
Supplement: sfag164_Supplemental_File [file sfag164_supplemental_file.docx]

Supplementary Materials

**Supplemental Figure S1**. Participant flow for the three outcome analyses

**Supplemental Figure S2.** Convergence diagnostics for multiple imputation of key CKD-MBD laboratory variables

**Supplemental Figure S3.** Observed versus imputed distributions of key laboratory variables

**Supplemental Figure S4**. Time courses of the CKD-MBD parameters

**Supplemental Figure S5**. Exploratory **s**tratified analysis for all-cause mortality

**Supplemental Figure S6**. Exploratory stratified analysis for CVD mortality

**Supplemental Figure S7**. Exploratory stratified analysis for bone fractures

**Supplemental Figure S8**. Exploratory stratified analysis for hip fractures

**Supplemental Figure S9.** Balance diagnostics for selected denominator-model covariates before and after MSM weighting (all-cause mortality cohort)

**Supplemental Figure S10.** Cumulative incidence of CVD death by baseline treatment category accounting for competing non-CVD death

**Supplemental Table S1.** Comparison of person-month records with observed versus missing i-PTH before multiple imputation

**Supplemental Table S2**. Multivariable logistic regression analysis of predictors of missing i-PTH before multiple imputation

**Supplemental Table S3**. Comparison of included and excluded person-month records due to missing VDRA or calcimimetic exposure data

**Supplemental Table S4**. Baseline characteristics of study patients in CVD mortality analysis.

**Supplemental Table S5**. Baseline characteristics of study patients in fracture analysis.

**Supplemental Table S6**. Number of patients in each treatment category over time

**Supplemental Table S7**. Drug continuation rates by follow-up period

**Supplemental Table S8.** Distribution of stabilized time-point and cumulative weights by treatment category

**Supplemental Table S9.** Indication-restricted sensitivity analysis of all-cause mortality among patients with baseline i-PTH ≥300 pg/mL

**Supplemental Table S10.** Complementary Fine–Gray subdistribution hazard analysis for CVD mortality using baseline treatment category

**Supplemental Figure S1**. Participant flow for the three outcome analyses


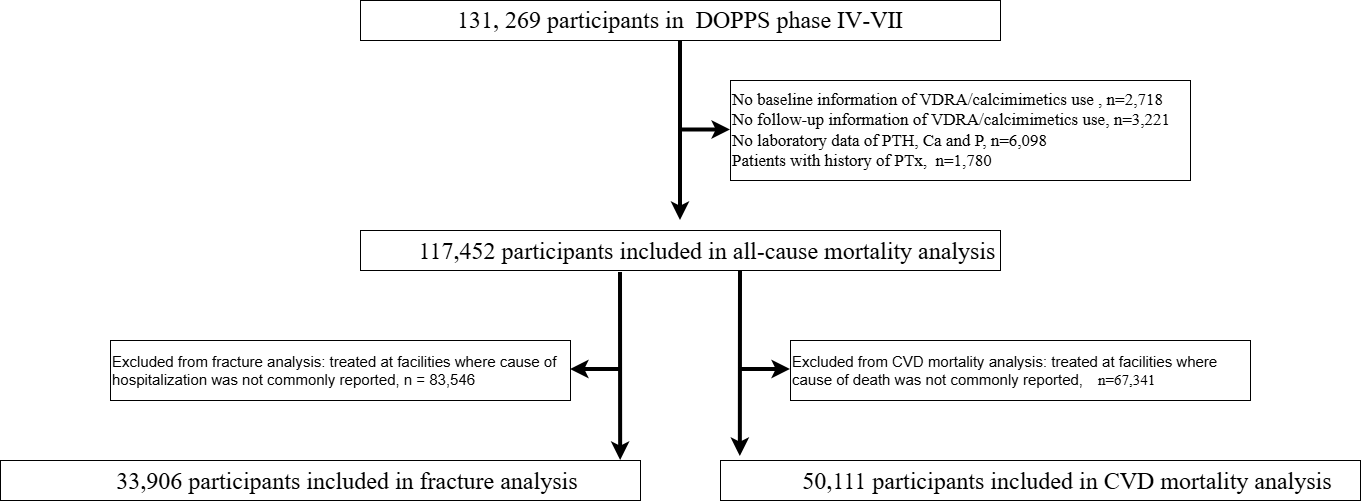


**Abbreviations:** Ca, serum calcium; CVD, cardiovascular disease; DOPPS, Dialysis Outcomes and Practice Patterns Study; P, serum phosphorus; PTH, parathyroid hormone; PTx, parathyroidectomy; VDRA, vitamin D-receptor activator.

For CVD mortality, the analytic cohort was restricted to facilities with commonly reported cause-of-death information; for fracture analyses, it was restricted to facilities with commonly reported cause-of-hospitalization information.

**Supplemental Figure S2.** Convergence diagnostics for multiple imputation of key CKD-MBD laboratory variables

**
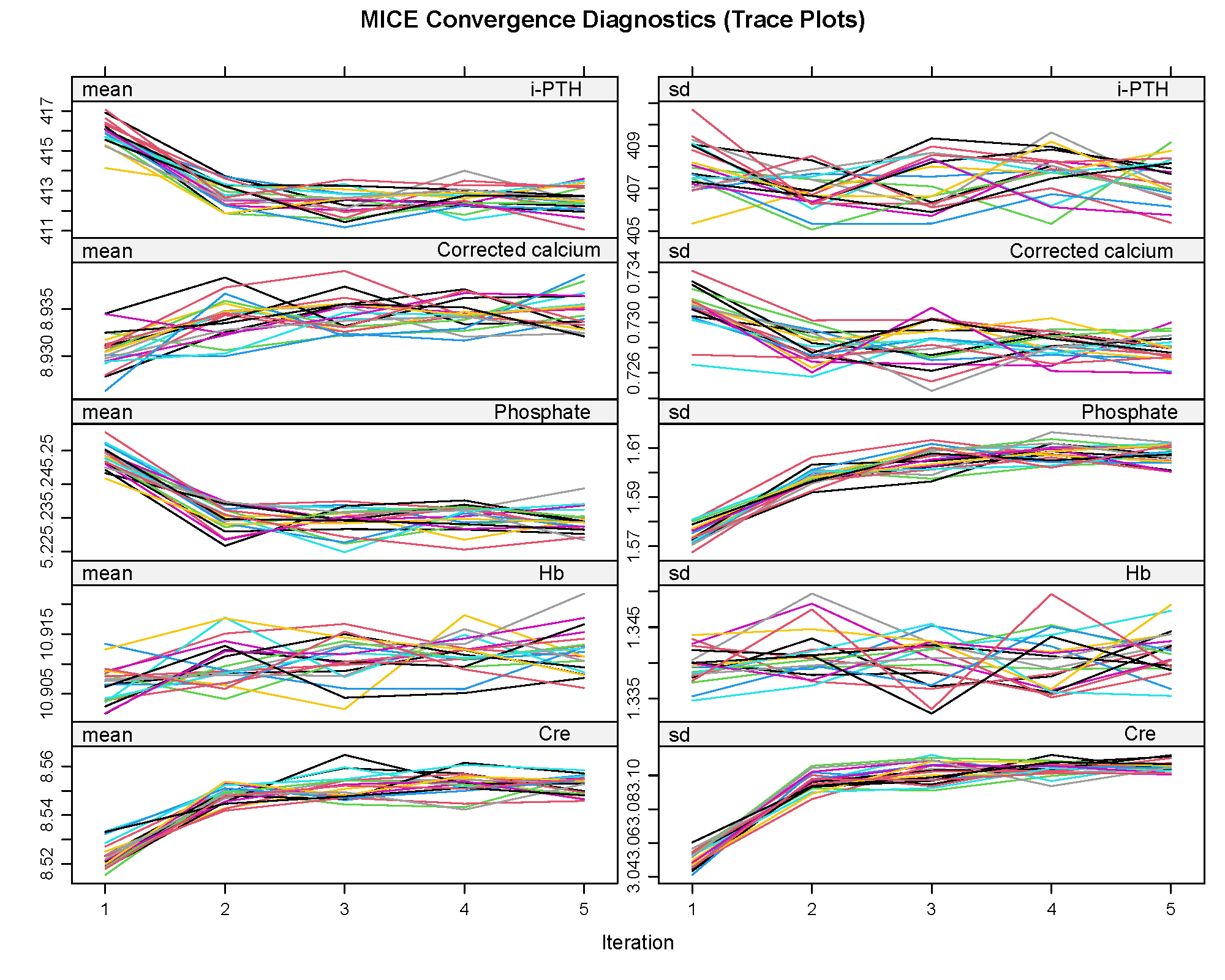
**

Trace plots from the multiple imputation by chained equations (MICE) procedure showing the mean and standard deviation of the imputed values across iterations for intact parathyroid hormone (-iPTH), serum calcium, serum phosphate, serum hemoglobin, and serum creatinine. Twenty imputed datasets were generated. The absence of meaningful systematic drift across iterations indicates adequate convergence of the imputation procedure.

**Abbreviations:** CKD-MBD, chronic kidney disease–mineral and bone disorder; i-PTH, intact parathyroid hormone; MICE, multiple imputation by chained equations; Hb, hemoglobin; Cre, Creatinine.

**
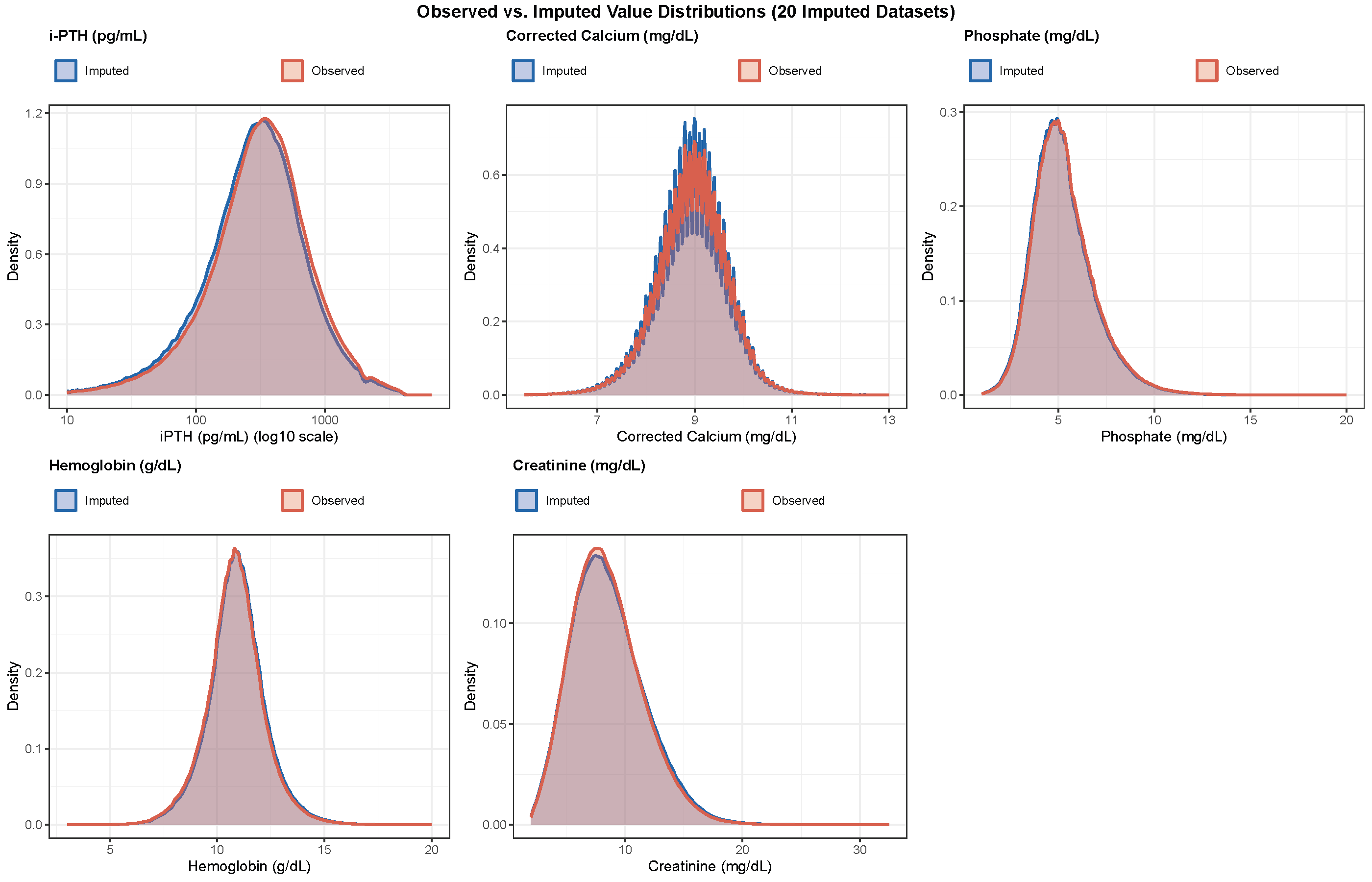
Supplemental Figure S3.** Observed versus imputed distributions of key laboratory variables

Density plots comparing observed values and imputed values obtained from the 20 MICE datasets for intact parathyroid hormone (i-PTH), serum calcium, serum phosphate, hemoglobin, and serum creatinine. The overall similarity between observed and imputed distributions supports the plausibility of the imputation model.

**Abbreviations:** i-PTH, intact parathyroid hormone; MICE, multiple imputation by chained equations.


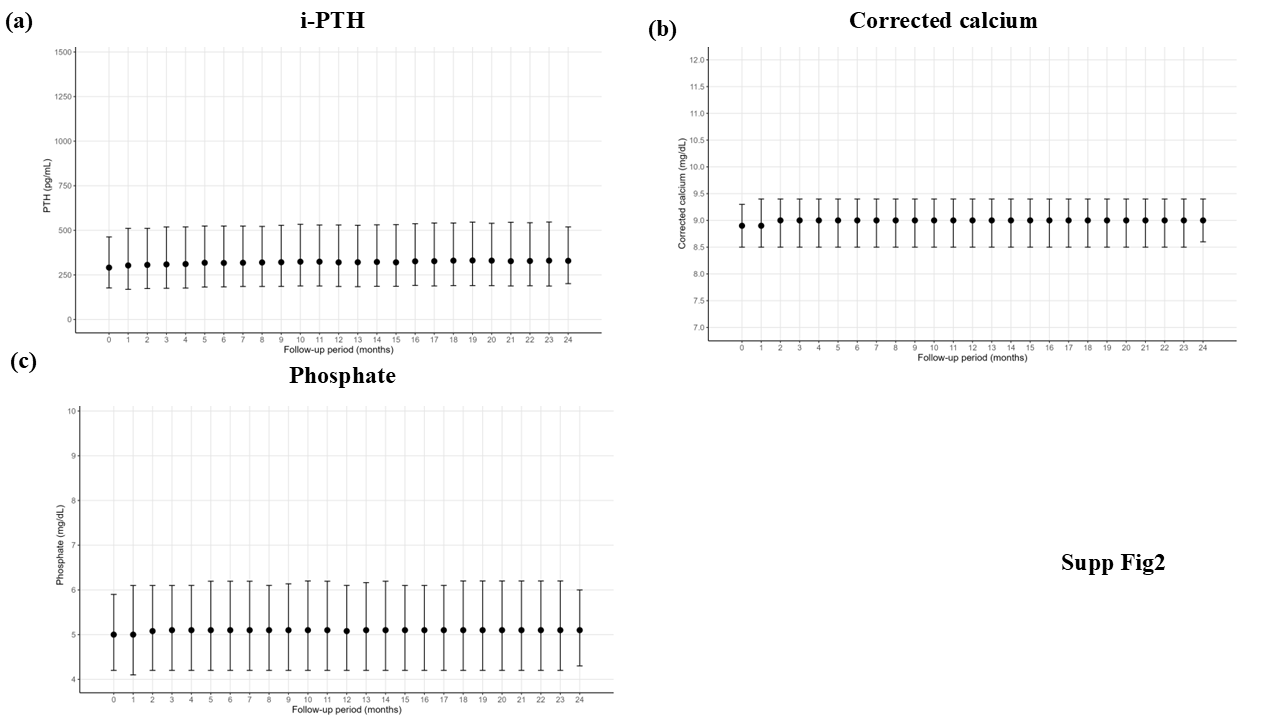
**Supplemental Figure S4**. Time courses of the CKD-MBD parameters

Panels show monthly distributions of the three principal laboratory markers of chronic kidney disease–mineral and bone disorder (CKD-MBD). The x-axis is follow-up time (months, 0–24) in all graphs.

(a) Intact parathyroid hormone (i-PTH).

(b) Albumin-corrected serum calcium.

(c) Serum phosphate.

For every month, a solid black circle marks the median value of the marker in the analytic cohort, and the accompanying solid black vertical bar represents the inter-quartile range (IQR; 25th–75th percentile).

**Abbreviations:** CKD-MBD, chronic kidney disease–mineral and bone disorder; i-PTH, intact parathyroid hormone; IQR, inter-quartile range.

**
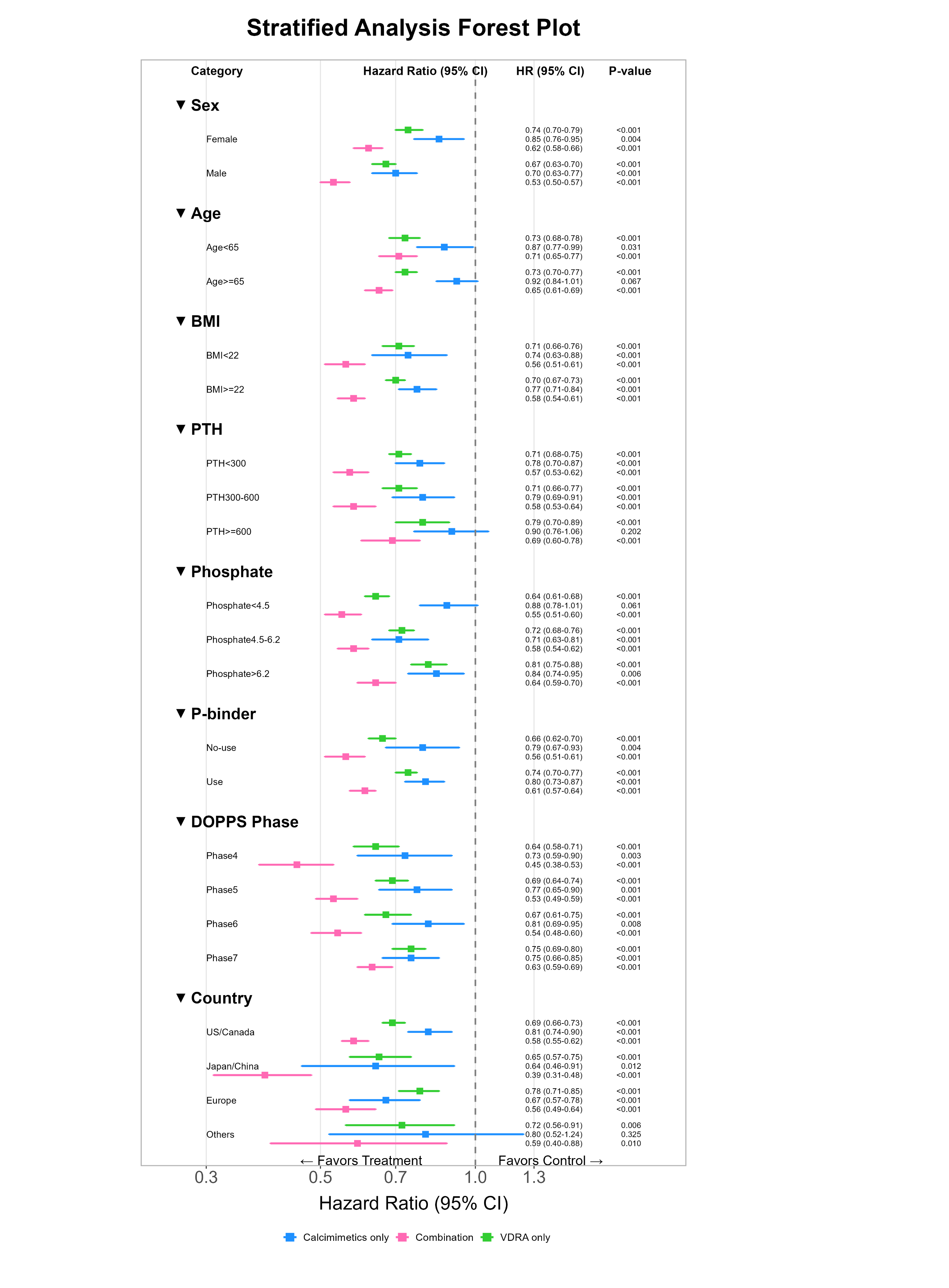
Supplemental Figure S5.** Exploratory stratified analysis for all-cause mortality

The plot displays adjusted hazard ratios (HRs) with 95 % confidence intervals (horizontal error bars) for all-cause death obtained from marginal-structural Cox models. Estimates are shown separately for each baseline characteristic listed on the y-axis. Combination therapy; pink,

VDRA only; green,

Calcimimetic only; blue

Reference category Patients receiving neither agent (vertical dashed line at HR = 1.0).

Abbreviations: BMI, body mass index; CI, confidence interval; DOPPS, Dialysis Outcomes and Practice Patterns Study; HR, hazard ratio; PTH, parathyroid hormone; VDRA, vitamin D receptor activator.

**
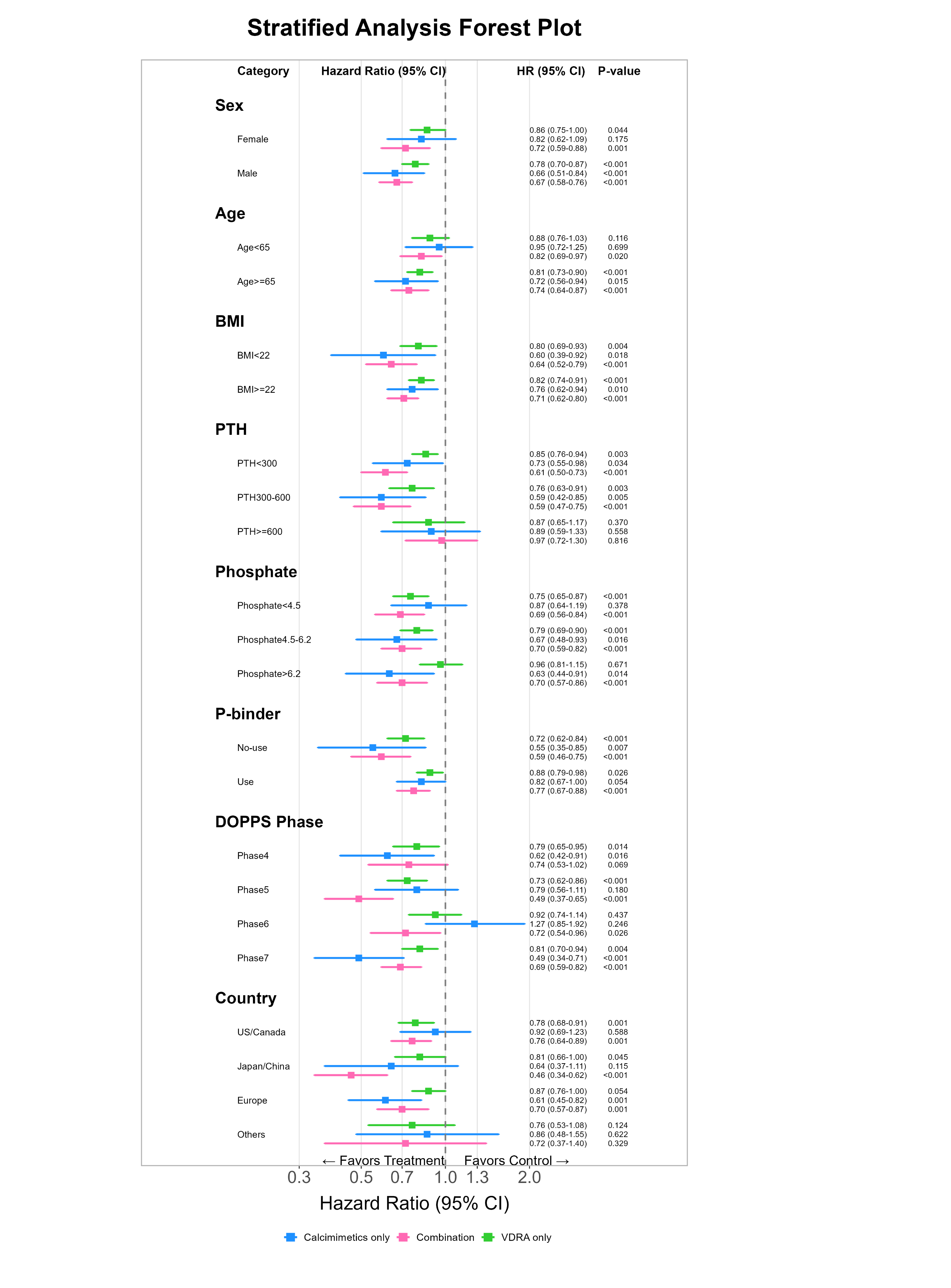
Supplemental Figure S6.** Exploratory stratified analysis for CVD mortality

The plot displays adjusted hazard ratios (HRs) with 95 % confidence intervals (horizontal error bars) for CVD death obtained from marginal-structural Cox models. Estimates are shown separately for each baseline characteristic listed on the y-axis. Combination therapy; pink,

VDRA only; green,

Calcimimetic only; blue

Reference category Patients receiving neither agent (vertical dashed line at HR = 1.0).

Abbreviations: BMI, body mass index; CI, confidence interval; DOPPS, Dialysis Outcomes and Practice Patterns Study; HR, hazard ratio; PTH, parathyroid hormone; VDRA, vitamin D receptor activator.

**
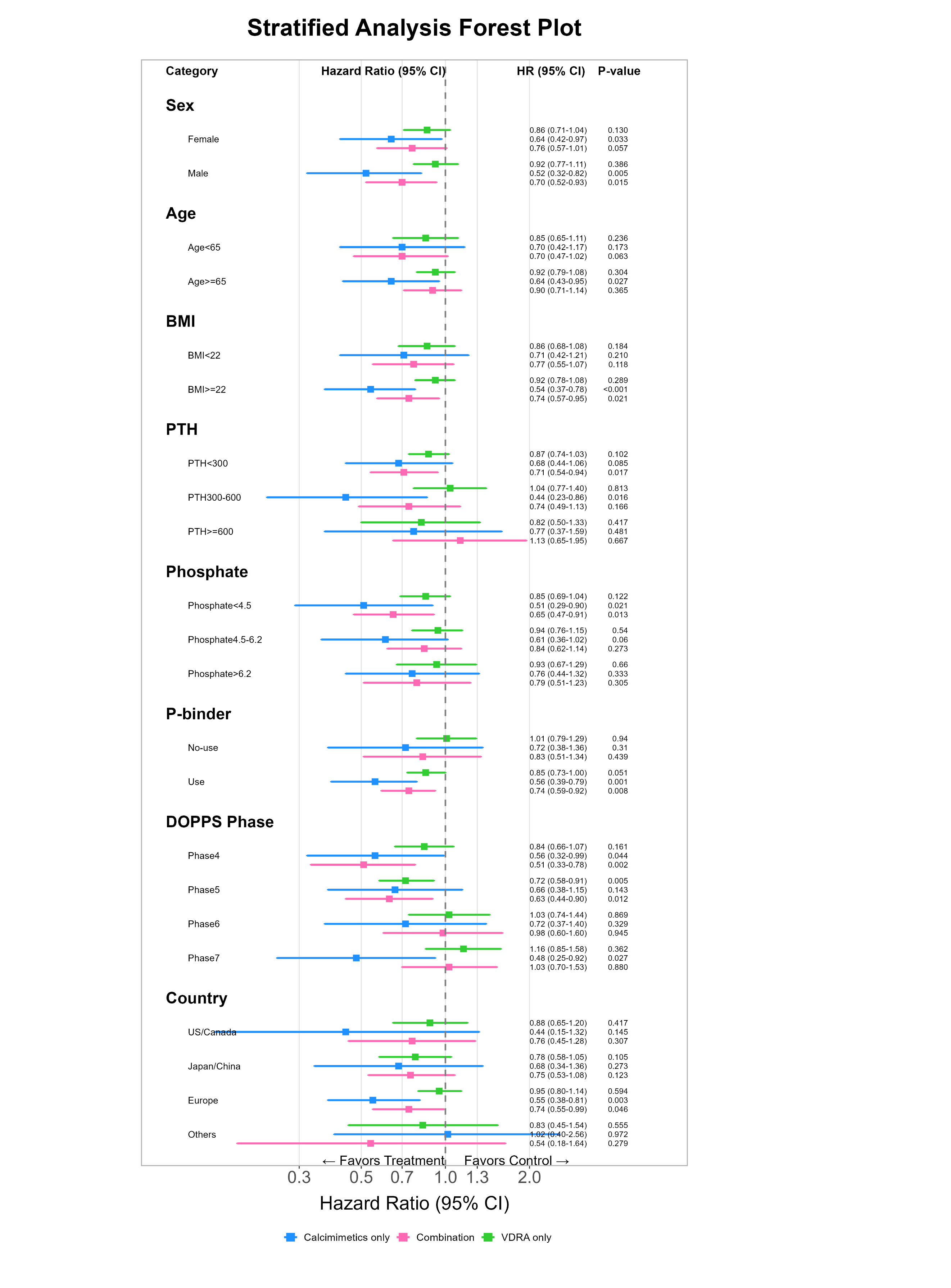
Supplemental Figure S7.** Exploratory stratified analysis for bone fractures

The plot displays adjusted hazard ratios (HRs) with 95 % confidence intervals (horizontal error bars) for bone fractures obtained from marginal-structural Cox models. Estimates are shown separately for each baseline characteristic listed on the y-axis.

Combination therapy; pink,

VDRA only; green,

Calcimimetic only; blue

Reference category Patients receiving neither agent (vertical dashed line at HR = 1.0).

**Abbreviations**: BMI, body mass index; CI, confidence interval; DOPPS, Dialysis Outcomes and Practice Patterns Study; HR, hazard ratio; PTH, parathyroid hormone; VDRA, vitamin D receptor activator.

**
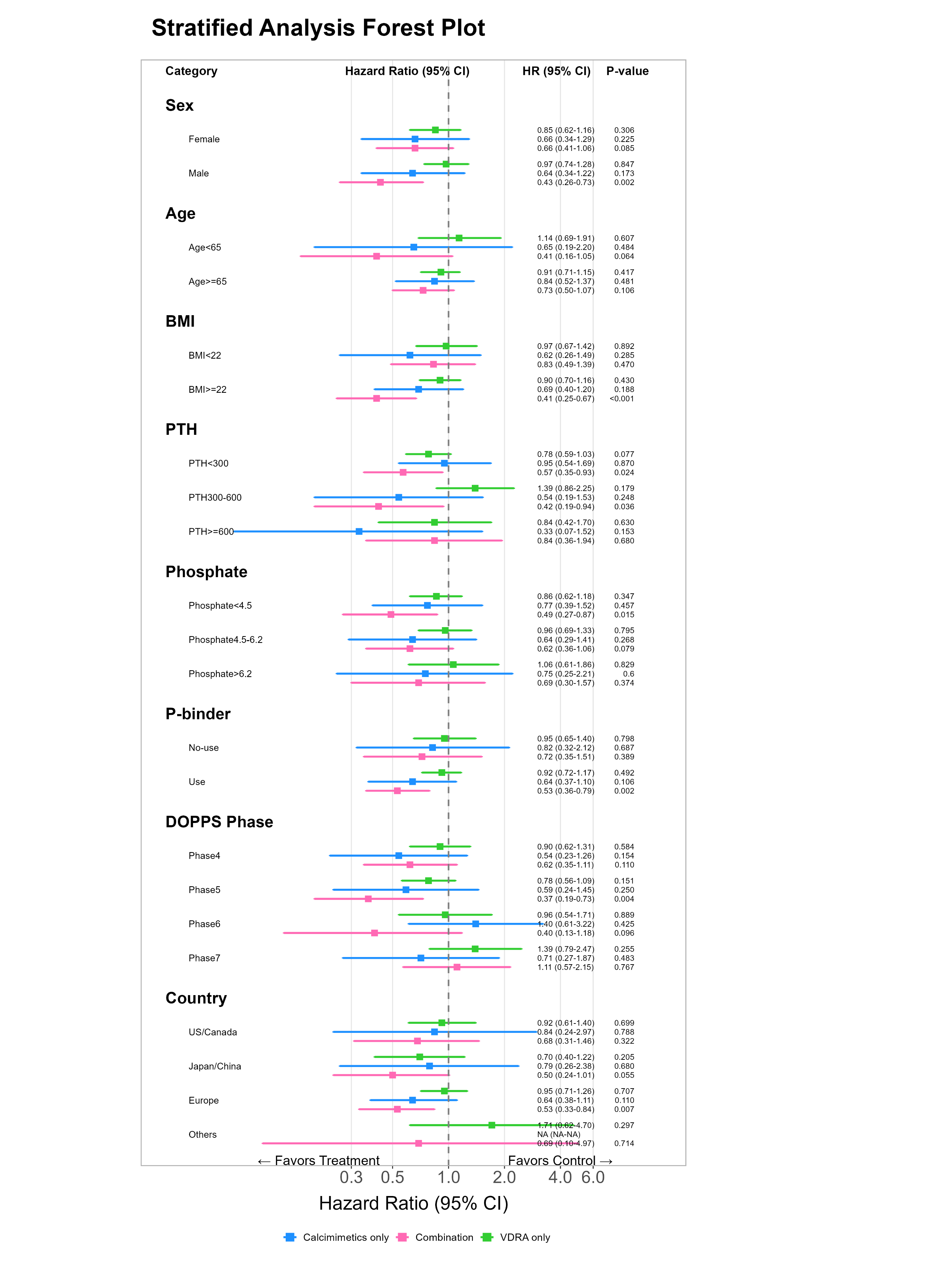
Supplemental Figure S8.** Exploratory Stratified analysis for hip fractures

The plot displays adjusted hazard ratios (HRs) with 95 % confidence intervals (horizontal error bars) for hip fractures obtained from marginal-structural Cox models. Estimates are shown separately for each baseline characteristic listed on the y-axis.

Combination therapy; pink,

VDRA only; green,

Calcimimetic only; blue

Reference category Patients receiving neither agent (vertical dashed line at HR = 1.0).

Abbreviations: BMI, body mass index; CI, confidence interval; DOPPS, Dialysis Outcomes and Practice Patterns Study; HR, hazard ratio; PTH, parathyroid hormone; VDRA, vitamin D receptor activator.

**Supplemental Figure S9.** Balance diagnostics for selected denominator-model covariates before and after MSM weighting (all-cause mortality cohort)

**
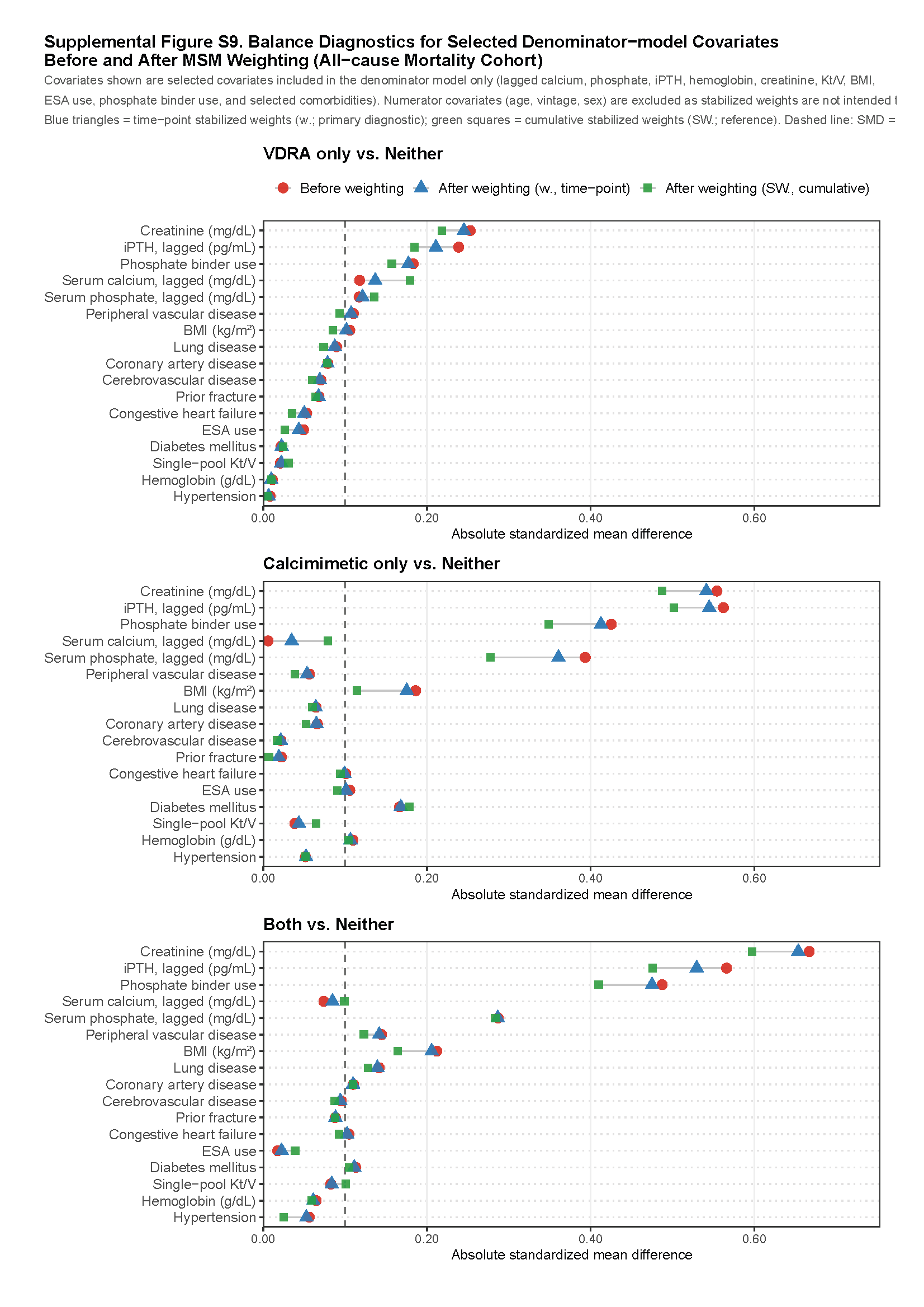
**

Covariates shown are selected covariates included in the denominator model. Because stabilized weights preserve numerator-model

covariates and are not intended to balance them, age, sex, and dialysis vintage were excluded from this balance panel. Red circles =

before weighting; blue triangles = after time-point stabilized weighting (w_t; primary diagnostic); green squares = after cumulative

stabilized weighting (SW_t; reference). The dashed line indicates SMD = 0.10.

**Abbreviations:** BMI, body mass index; ESA, erythropoiesis-stimulating agent; iPTH, intact parathyroid hormone; SMD, standardized mean difference; SW_t, cumulative stabilized weight; w_t, time-point stabilized weight.

**Supplemental Figure S10.** Cumulative incidence of CVD death by baseline treatment category accounting for competing non-CVD death

**
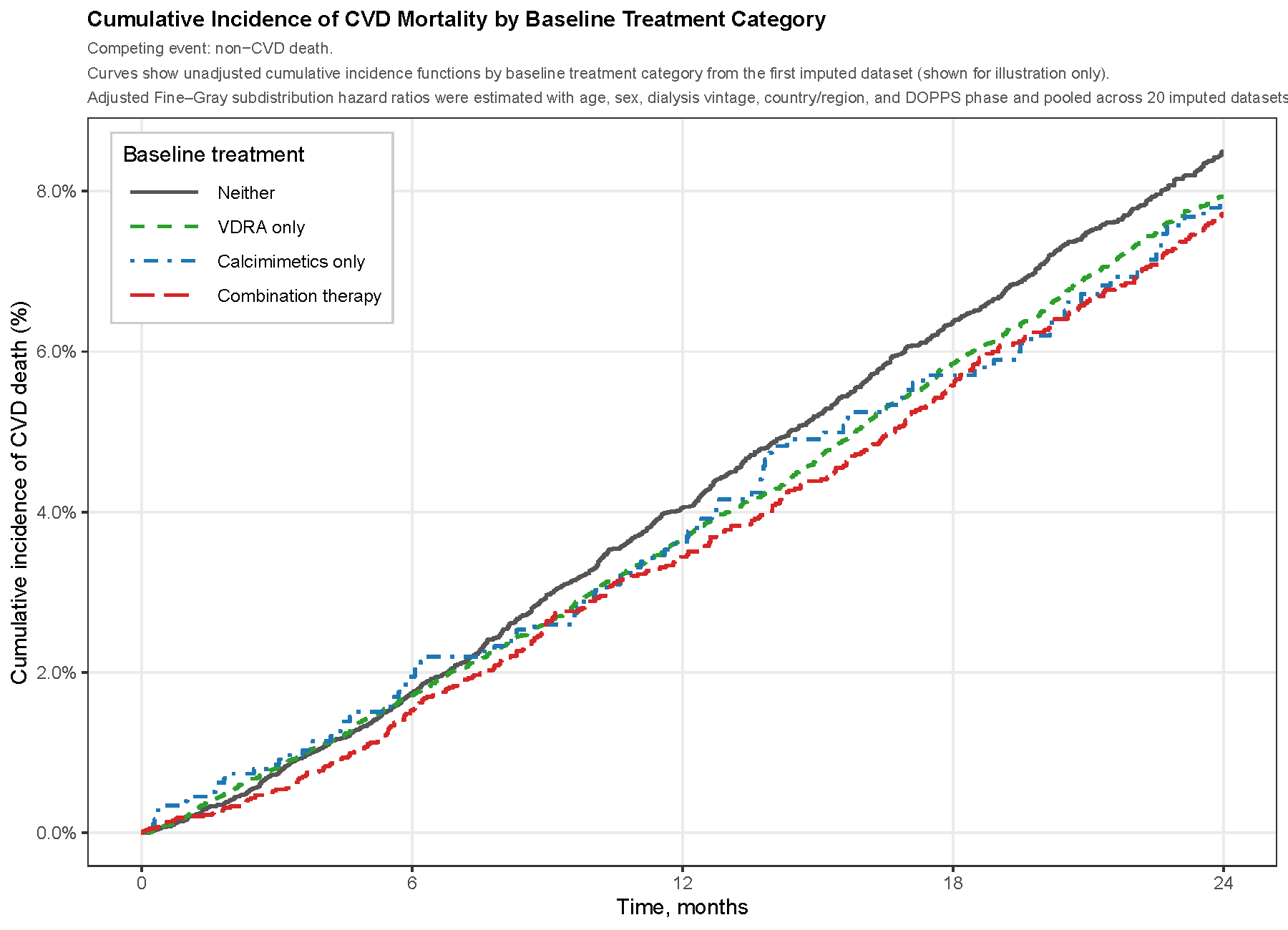
**

Curves show unadjusted cumulative incidence functions for CVD death according to baseline treatment category (neither use, VDRA

only, calcimimetics only, or combination therapy), with non-CVD death treated as the competing event. Curves are shown from the

first imputed dataset for illustration. Adjusted subdistribution hazard ratios were estimated using Fine–Gray models including age,

sex, dialysis vintage, country/region, and DOPPS phase and were pooled across 20 multiply imputed datasets using Rubin’s rules

(**Supplemental Table S10**). Because this complementary analysis used baseline treatment category, it provides a cumulative incidence perspective and does not replace the primary monthly time-updated cause-specific MSM analysis.

**Abbreviations:** CVD, cardiovascular disease; SHR, subdistribution hazard ratio; VDRA, vitamin D receptor activator.

**Supplemental Table S1.** Comparison of person-month records with observed versus missing i-PTH before multiple imputation

| **Characteristics** | | **Overall** | **Missing i-PTH** | **Observed i-PTH** | **SMD** |
| --- | --- | --- | --- | --- | --- |
| **Number** | | 1895467 | 750696 | 1144771 |  |
| **Age** |  | 63.6 (14.4) | 64.1 (14.4) | 63.2 (14.5) | 0.066 |
| **Male sex** |  | 1107388 (58.4) | 446645 (59.5) | 660743 (57.7) | 0.036 |
| **Race (Black)** |  | 463250 (26.6) | 138353 (19.7) | 324897 (31.2) | 0.266 |
| **Country** |  |  |  |  | 0.384 |
|  | **US/Canada** | 1392264 (73.5) | 477269 (63.6) | 914995 (79.9) |  |
|  | **Japan/China** | 222061 (11.7) | 108667 (14.5) | 113394 (9.9) |  |
|  | **Europe^#1^** | 253021 (13.3) | 150586 (20.1) | 102435 (8.9) |  |
|  | **Others^#2^** | 28121 (1.5) | 14174 (1.9) | 13947 (1.2) |  |
| **Phase** |  |  |  |  | 0.165 |
|  | **Ⅳ** | 233259 (12.3) | 113999 (15.2) | 119260 (10.4) |  |
|  | **Ⅴ** | 487026 (25.7) | 200439 (26.7) | 286587 (25.0) |  |
|  | **Ⅵ** | 457151 (24.1) | 176345 (23.5) | 280806 (24.5) |  |
|  | **Ⅶ** | 718031 (37.9) | 259913 (34.6) | 458118 (40.0) |  |
| **HD vintage (years)** |  | 8.9 (0.7) | 8.9 (0.7) | 9.0 (0.7) | 0.029 |
| **BMI (kg/m^2^)** |  | 27.6 (7.0) | 27.2 (6.9) | 27.9 (7.1) | 0.102 |
| **Sp Kt/V** |  | 1.6 (0.3) | 1.6 (0.3) | 1.6 (0.3) | 0.025 |
| ***Comorbidities*** |  |  |  |  |  |
|  | **Diabetes** | 1043002 (54.3) | 405152 (52.6) | 637850 (55.5) | 0.058 |
|  | **Hypertension** | 1483216 (77.2) | 602979 (78.2) | 880237 (76.5) | 0.041 |
|  | **Coronary heart disease** | 429560 (22.4) | 189647 (24.6) | 239913 (20.9) | 0.089 |
|  | **Congestive heart failure** | 402289 (20.9) | 161494 (21.0) | 240795 (20.9) | 0.001 |
|  | **Other Cardiovascular disease** | 362110 (18.9) | 157048 (20.4) | 205062 (17.8) | 0.065 |
|  | **Cerebrovascular disease** | 169019 (8.8) | 75419 (9.8) | 93600 (8.1) | 0.058 |
|  | **Peripheral vascular disease** | 279961 (14.6) | 126801 (16.5) | 153160 (13.3) | 0.088 |
|  | **Cancer** | 150549 (7.8) | 69290 (9.0) | 81259 (7.1) | 0.071 |
|  | **Neurologic disease** | 118922 (6.2) | 52804 (6.9) | 66118 (5.7) | 0.045 |
|  | **Lung disease** | 144493 (7.5) | 62332 (8.1) | 82161 (7.1) | 0.036 |
|  | **History of hip/vertebral fractures** | 28704 (1.5) | 15919 (2.1) | 12785 (1.1) | 0.076 |
| ***Laboratory*** |  |  |  |  |  |
|  | **Hemoglobin (g/dL)** | 10.8 (1.3) | 10.9 (1.3) | 10.8 (1.3) | 0.059 |
|  | **BUN (mg/dL)** | 56.6 (18.8) | 56.8 (18.9) | 56.5 (18.6) | 0.011 |
|  | **Creatinine (mg/dL)** | 8.5 (3.0) | 8.4 (3.0) | 8.5 (3.0) | 0.05 |
|  | **Albumin-corrected calcium (mg/dL)** | 8.9 (0.7) | 8.9 (0.7) | 9.0 (0.7) | 0.033 |
|  | **Phosphorus (mg/dL)** | 5.3 (1.6) | 5.2 (1.6) | 5.3 (1.6) | 0.063 |
| ***Medications*** |  |  |  |  |  |
|  | **ESA use** | 1645605 (87.5) | 647680 (87.0) | 997925 (87.9) | 0.028 |
|  | **Phosphate-binder use** | 1491337 (78.8) | 582367 (77.7) | 908970 (79.5) | 0.043 |

Characteristics of person-month records with observed i-PTH and those with missing i-PTH in the original dataset before multiple imputation. Values are presented as mean (standard deviation) or number (percentage). Standardized mean differences (SMDs) are shown to quantify the magnitude of between-group differences. Because of the large sample size, formal hypothesis tests were not emphasized for this descriptive comparison. Race (Black) was coded as Black versus non-Black.

#1 Europe includes Belgium, France, Germany, Italy, Spain, Sweden, and the United Kingdom.

#2 Others includes Australia/New Zealand, Gulf Cooperation Council countries, Russia, and Turkey.

**Abbreviations**: BUN, blood urea nitrogen; ESA, erythropoiesis-stimulating agent; i-PTH, intact parathyroid hormone; spKt/V, single-pool Kt/V; SMD, standardized mean difference; VDRA, vitamin D receptor activator.

**Supplemental Table S2**. Multivariable logistic regression analysis of predictors of missing i-PTH before multiple imputation

| **Characteristics** | | **Adjusted OR (95% CI)** | **p-value** |
| --- | --- | --- | --- |
| **Age (per 10 years)** |  | 1.00 (0.99–1.00) | 0.133 |
| **Male sex (female as reference)** |  | 1.01 (1.00–1.02) | 0.035 |
| **Race (Black) (Non-Black as reference)** |  | 0.74 (0.73–0.75) | <0.001 |
| **Country (US/Canada as reference)** |  |  |  |
|  | **Japan/China** | 1.65 (1.62–1.67) | <0.001 |
|  | **Europe^#1^** | 1.39 (1.37–1.41) | <0.001 |
|  | **Others^#2^** | 0.99 (0.95–1.03) | 0.604 |
| **Phase (Phase IV as reference)** |  |  |  |
|  | **Ⅴ** | 0.94 (0.93–0.95) | <0.001 |
|  | **Ⅵ** | 0.72 (0.71–0.73) | <0.001 |
|  | **Ⅶ** | 0.60 (0.59–0.61) | <0.001 |
| **HD vintage (per 5 years)** |  | 1.00 (1.00–1.01) | 0.234 |
| **BMI (per 5 kg/m²)** |  | 1.00 (1.00–1.00) | 0.993 |
| **Sp Kt/V(per 0.1 unit)** |  | 1.01 (1.00–1.01) | <0.001 |
| ***Comorbidities*** |  |  |  |
|  | **Diabetes** | 0.98 (0.97–0.99) | <0.001 |
|  | **Hypertension** | 1.03 (1.02–1.04) | <0.001 |
|  | **Coronary heart disease** | 1.03 (1.01–1.04) | <0.001 |
|  | **Congestive heart failure** | 0.98 (0.97–0.99) | <0.001 |
|  | **Other Cardiovascular disease** | 1.01 (1.00–1.02) | 0.146 |
|  | **Cerebrovascular disease** | 1.01 (0.99–1.02) | 0.530 |
|  | **Peripheral vascular disease** | 1.03 (1.02–1.05) | <0.001 |
|  | **Cancer** | 1.00 (0.98–1.02) | 0.954 |
|  | **Neurologic disease** | 0.98 (0.96–1.00) | 0.013 |
|  | **Lung disease** | 0.94 (0.93–0.96) | <0.001 |
|  | **History of hip/vertebral fractures** | 1.03 (0.99–1.06) | 0.176 |
| ***Laboratory*** |  |  |  |
|  | **Hemoglobin (g/dL)** | 1.03 (1.03–1.03) | <0.001 |
|  | **BUN (mg/dL)** | 1.00 (1.00–1.00) | <0.001 |
|  | **Creatinine (mg/dL)** | 1.00 (1.00–1.00) | 0.559 |
|  | **Albumin-corrected calcium (mg/dL)** | 0.96 (0.95–0.96) | <0.001 |
|  | **Phosphorus (mg/dL)** | 0.98 (0.98–0.98) | <0.001 |
| ***Medications*** |  |  |  |
|  | **ESA use** | 0.98 (0.97–0.99) | 0.004 |
|  | **Phosphate-binder use** | 1.00 (0.98–1.01) | 0.392 |
| ***Treatment (neither VDRA nor calcimimetic as reference)*** |  |  |  |
|  | **VDRA only** | 0.69 (0.69–0.70) | <0.001 |
|  | **Calcimimetics only** | 0.45 (0.44–0.46) | <0.001 |
|  | **Combination therapy** | 0.39 (0.38–0.39) | <0.001 |
| ***Model fit*** |  |  |  |
|  | **AUC (C-statistic)** | 0.650 |  |

Adjusted odds ratios (ORs) and 95% confidence intervals (CIs) from a multivariable logistic regression model in which the dependent variable was missingness of i-PTH.

The outcome variable was i-PTH missingness (1 = missing, 0 = observed). Odds ratios greater than 1 indicate higher odds of i-PTH being missing. The model was fitted to person-month records with complete data for the covariates included in the model. Treatment category was defined at each person-month record as neither VDRA nor calcimimetic, VDRA only, calcimimetics only, or combination therapy. Reference categories were female sex, non-Black race, US/Canada, DOPPS Phase IV, and neither VDRA nor calcimimetic use. The C-statistic denotes the area under the receiver operating characteristic curve.
#1 Europe includes Belgium, France, Germany, Italy, Spain, Sweden, and the United Kingdom.
#2 Others includes Australia/New Zealand, Gulf Cooperation Council countries, Russia, and Turkey.
**Abbreviations**: BMI, body mass index; BUN, blood urea nitrogen; CI, confidence interval; ESA, erythropoiesis-stimulating agent; i-PTH, intact parathyroid hormone; OR, odds ratio; spKt/V, single-pool Kt/V; VDRA, vitamin D receptor activator.

**Supplemental Table S3**. Comparison of included and excluded person-month records due to missing VDRA or calcimimetic exposure data

| **Summary** | | - **Total potential person-month records: 1,920,766** - **Excluded due to missing exposure: 25,299 (1.3%)** - **Total potential person-months: 2,018,827** - **Excluded person-months: 32,664 (1.6%)** - **Patients contributing at least one excluded month: 6,105/117,452 (5.2%)** | | | |
| --- | --- | --- | --- | --- | --- |
|  | |  | | | |
| **Characteristics** | | **Overall** | **Included** | **Excluded (missing exposure)** | **SMD** |
| **Number** | | 1920766 | 1895467 | 25299 |  |
| **Age** |  | 63.6 (14.4) | 63.6 (14.4) | 65.0 (14.5) | 0.099 |
| **Male sex** |  | 1123052 (58.5) | 1107388 (58.4) | 15664 (61.9) | 0.071 |
| **Race (Black)** |  | 463250 (26.6) | 461713 (26.9) | 1537 (6.5) | 0.568 |
| **Country** |  |  |  |  | 1.256 |
|  | **US/Canada** | 1398062 (72.8) | 1392264 (73.5) | 5798 (22.9) |  |
|  | **Japan/China** | 225680 (11.7) | 222061 (11.7) | 3619 (14.3) |  |
|  | **Europe^#1^** | 266409 (13.9) | 253021 (13.3) | 13388 (52.9) |  |
|  | **Others^#2^** | 30615 (1.6) | 28121 (1.5) | 2494 (9.9) |  |
| **Phase** |  |  |  |  | 0.355 |
|  | **Ⅳ** | 236372 (12.3) | 233259 (12.3) | 3113 (12.3) |  |
|  | **Ⅴ** | 492443 (25.6) | 487026 (25.7) | 5417 (21.4) |  |
|  | **Ⅵ** | 467178 (24.3) | 457151 (24.1) | 10027 (39.6) |  |
|  | **Ⅶ** | 724773 (37.7) | 718031 (37.9) | 6742 (26.6) |  |
| **HD vintage (years)** |  | 3.9 (4.8) | 3.9 (4.8) | 4.3 (5.4) | 0.069 |
| **BMI (kg/m^2^)** |  | 27.6 (7.0) | 27.6 (7.0) | 25.8 (6.4) | 0.269 |
| **Sp Kt/V** |  | 1.6 (0.3) | 1.6 (0.3) | 1.5 (0.3) | 0.10 |
| ***Comorbidities*** |  |  |  |  |  |
|  | **Diabetes** | 1043002 (54.3) | 1031788 (54.4) | 11214 (44.3) | 0.203 |
|  | **Hypertension** | 1483216 (77.2) | 1462025 (77.1) | 21191 (83.8) | 0.168 |
|  | **Coronary heart disease** | 429560 (22.4) | 421864 (22.3) | 7696 (30.4) | 0.186 |
|  | **Congestive heart failure** | 402289 (20.9) | 397338 (21.0) | 4951 (19.6) | 0.035 |
|  | **Other Cardiovascular disease** | 362110 (18.9) | 355369 (18.7) | 6741 (26.6) | 0.189 |
|  | **Cerebrovascular disease** | 169019 (8.8) | 165593 (8.7) | 3426 (13.5) | 0.153 |
|  | **Peripheral vascular disease** | 279961 (14.6) | 274284 (14.5) | 5677 (22.4) | 0.207 |
|  | **Cancer** | 150549 (7.8) | 147422 (7.8) | 3127 (12.4) | 0.153 |
|  | **Neurologic disease** | 118922 (6.2) | 116429 (6.1) | 2493 (9.9) | 0.137 |
|  | **Lung disease** | 144493 (7.5) | 141558 (7.5) | 2935 (11.6) | 0.141 |
|  | **History of hip/vertebral fractures** | 28704 (1.5) | 27437 (1.4) | 1267 (5.0) | 0.202 |
| ***Laboratory*** |  |  |  |  |  |
|  | **Hemoglobin (g/dL)** | 10.8 (1.3) | 10.8 (1.3) | 11.2 (1.4) | 0.248 |
|  | **BUN (mg/dL)** | 56.6 (18.8) | 56.6 (18.8) | 56.1 (18.9) | 0.031 |
|  | **Creatinine (mg/dL)** | 8.5 (3.0) | 8.5 (3.0) | 8.1 (3.0) | 0.127 |
|  | **Albumin-corrected calcium (mg/dL)** | 8.9 (0.7) | 8.9 (0.7) | 9.0 (0.7) | 0.041 |
|  | **Phosphorus (mg/dL)** | 5.3 (1.6) | 5.3 (1.6) | 5.0 (1.6) | 0.201 |
| ***Medications*** |  |  |  |  |  |
|  | **ESA use** | 1651216 (87.5) | 1645605 (87.5) | 5611 (87.6) | 0.001 |
|  | **Phosphate-binder use** | 1495348 (78.8) | 1491337 (78.8) | 4011 (88.0) | 0.25 |

Summary statistics are shown in the upper panel. “Person-month records” refers to observation intervals in the counting-process dataset, whereas “person-months” refers to the summed duration of those intervals. Values in the lower panel are presented as mean (standard deviation) or number (percentage). Standardized mean differences (SMDs) are shown to quantify the magnitude of differences between included and excluded person-month records. Patients were not mutually exclusive between groups, because an individual patient could contribute both included and excluded person-month records during follow-up.
#1 Europe includes Belgium, France, Germany, Italy, Spain, Sweden, and the United Kingdom.
#2 Others includes Australia/New Zealand, Gulf Cooperation Council countries, Russia, and Turkey.
**Abbreviations**: BUN, blood urea nitrogen; ESA, erythropoiesis-stimulating agent; SMD, standardized mean difference; spKt/V, single-pool Kt/V; VDRA, vitamin D receptor activator.

**Supplemental Table S4. Baseline characteristics of study patients in CVD mortality analysis**

| **Characteristics** | | **All patients** | **Neither use** | **VDRA use** | **Calcimimetics use** | **Combination use** |
| --- | --- | --- | --- | --- | --- | --- |
| **Number** | | 50111 | 18033 | 24126 | 1873 | 6079 |
| **Age** |  | 65 [54, 75] | 67 [56, 76] | 65 [54, 75] | 63 [51, 73] | 61 [50, 71] |
| **Male sex** |  | 30531 (61.0) | 11017 (61.1) | 14847 (61.6) | 1091 (58.2) | 3576 (58.8) |
| **Race (%Black)** |  | 6399 (13.6) | 1063 (6.4) | 3666 (16.1) | 139 (8.0) | 1531 (26.0) |
| **Country** |  |  |  |  |  |  |
|  | **US/Canada** | 22032 (44.0) | 6477 (35.9) | 11607 (48.1) | 548 (29.3) | 3400 (55.9) |
|  | **Japan/China** | 9381 (18.7) | 3367 (18.7) | 4684 (19.4) | 275 (14.7) | 1055 (17.4) |
|  | **Europe^#1^** | 15516 (31.0) | 6920 (38.4) | 6323 (26.2) | 912 (48.7) | 1361 (22.4) |
|  | **Others^#2^** | 3182 (6.3) | 1269 (7.0) | 1512 (6.3) | 138 (7.4) | 263 (4.3) |
| **Phase** |  |  |  |  |  |  |
|  | **Ⅳ** | 9601 (19.2) | 4157 (23.1) | 4322 (17.9) | 424 (22.6) | 698 (11.5) |
|  | **Ⅴ** | 13988 (27.9) | 5360 (29.7) | 6728 (27.9) | 474 (25.3) | 1426 (23.5) |
|  | **Ⅵ** | 11612 (23.2) | 3565 (19.8) | 5921 (24.5) | 425 (22.7) | 1701 (28.0) |
|  | **Ⅶ** | 14910 (29.8) | 4951 (27.5) | 7155 (29.7) | 550 (29.4) | 2254 (37.1) |
| **HD vintage (years)** |  | 1.99 [0.41, 5.15] | 0.99 [0.28, 3.35] | 1.95 [0.42, 4.77] | 4.55 [2.04, 8.53] | 5.55 [2.86, 9.56] |
| **BMI (kg/m^2^)** |  | 25.39 [21.89, 30.06] | 24.93 [21.63, 29.35] | 25.52 [21.95, 30.19] | 25.46 [22.04, 30.13] | 26.20 [22.39, 31.34] |
| **Sp Kt/V** |  | 1.50 [1.30, 1.70] | 1.47 [1.26, 1.69] | 1.49 [1.30, 1.70] | 1.54 [1.35, 1.73] | 1.57 [1.40, 1.74] |
| ***Comorbidities*** |  |  |  |  |  |  |
|  | **Diabetes** | 25453 (50.8) | 9147 (50.7) | 12676 (52.5) | 765 (40.8) | 2865 (47.1) |
|  | **Hypertension** | 43238 (86.3) | 15398 (85.4) | 20796 (86.2) | 1648 (88.0) | 5396 (88.8) |
|  | **Coronary heart disease** | 14963 (29.9) | 5781 (32.1) | 6867 (28.5) | 573 (30.6) | 1742 (28.7) |
|  | **Congestive heart failure** | 10424 (20.8) | 4050 (22.5) | 4796 (19.9) | 374 (20.0) | 1204 (19.8) |
|  | **Other Cardiovascular disease** | 11844 (23.6) | 4549 (25.2) | 5363 (22.2) | 520 (27.8) | 1412 (23.2) |
|  | **Cerebrovascular disease** | 5947 (11.9) | 2424 (13.4) | 2690 (11.1) | 234 (12.5) | 599 (9.9) |
|  | **Peripheral vascular disease** | 10433 (20.8) | 4137 (22.9) | 4615 (19.1) | 441 (23.5) | 1240 (20.4) |
|  | **Cancer** | 5548 (11.1) | 2333 (12.9) | 2433 (10.1) | 217 (11.6) | 565 (9.3) |
|  | **Neurologic disease** | 4595 (9.2) | 1860 (10.3) | 2001 (8.3) | 167 (8.9) | 567 (9.3) |
|  | **Lung disease** | 4851 (9.7) | 2099 (11.6) | 2084 (8.6) | 193 (10.3) | 475 (7.8) |
|  | **History of hip/vertebral fractures** | 1617 (3.2) | 702 (3.9) | 697 (2.9) | 81 (4.3) | 137 (2.3) |
| ***Laboratory*** |  |  |  |  |  |  |
|  | **Hemoglobin (g/dL)** | 10.90 [10.00, 11.70] | 10.80 [9.80, 11.70] | 10.90 [10.10, 11.70] | 11.10 [10.20, 12.00] | 11.00 [10.20, 11.80] |
|  | **BUN (mg/dL)** | 55.00 [43.24, 68.00] | 54.00 [42.00, 67.67] | 55.00 [43.40, 68.00] | 58.80 [47.13, 71.40] | 57.00 [46.00, 69.00] |
|  | **Cre (mg/dL)** | 7.85 [5.93, 10.03] | 7.08 [5.26, 9.27] | 7.90 [6.07, 10.02] | 9.00 [7.11, 11.00] | 9.44 [7.62, 11.59] |
|  | **Ca (mg/dL)** | 8.90 [8.40, 9.36] | 8.84 [8.40, 9.30] | 8.90 [8.50, 9.40] | 8.88 [8.34, 9.40] | 8.94 [8.48, 9.40] |
|  | **Phosphorus (mg/dL)** | 5.00 [4.10, 6.07] | 4.80 [3.96, 5.90] | 5.00 [4.20, 6.00] | 5.20 [4.20, 6.66] | 5.26 [4.40, 6.40] |
|  | **i-PTH (pg/mL)** | 275.00 [151.00, 478.00] | 200.00 [111.00, 331.56] | 308.00 [176.80, 497.00] | 402.50 [219.25, 805.53] | 420.80 [221.52, 752.95] |
| ***Medications*** |  |  |  |  |  |  |
|  | **ESA use** | 42197 (85.6) | 14992 (84.7) | 20640 (86.8) | 1493 (80.9) | 5072 (84.6) |
|  | **Phosphate-binder use** | 36289 (72.8) | 11353 (63.6) | 18060 (75.1) | 1552 (83.6) | 5324 (87.9) |

Values are median [interquartile range], or number (proportion).

Abbreviations: BMI, body mass index; BUN, blood urea nitrogen; Ca, albumin-corrected serum calcium; Cre, serum creatinine; ESA, erythropoiesis-stimulating agent; HD vintage, years since initiation of maintenance haemodialysis; i-PTH, intact parathyroid hormone; spKt/V, single-pool Kt/V (dialysis adequacy index); SMD, standardised mean difference; VDRA, vitamin D receptor activator.

#1 Europe: Belgium, France, Germany, Italy, Spain, Sweden and UK

#2 Others: Australia-New Zealand, GCC, Russia and Turkey

**Supplemental Table S5. Baseline characteristics of study patients in fracture analysis**

| **Characteristics** | | **All patients** | **Neither use** | **VDRA use** | **Calcimimetics use** | **Combination use** |
| --- | --- | --- | --- | --- | --- | --- |
| **Number** | | 33906 | 14057 | 15131 | 1554 | 3164 |
| **Age** |  | 66.89 [55.00, 76.00] | 67.56 [56.00, 77.00] | 67.00 [55.00, 76.00] | 64.00 [52.00, 74.00] | 63.00 [52.54, 73.00] |
| **Male sex** |  | 21232 (62.7) | 8795 (62.6) | 9584 (63.4) | 939 (60.4) | 1914 (60.5) |
| **Race (%Black)** |  | 1194 (3.8) | 332 (2.6) | 677 (4.7) | 41 (2.8) | 144 (4.7) |
| **Country** |  |  |  |  |  |  |
|  | **US/Canada** | 4962 (14.6) | 2119 (15.1) | 2324 (15.4) | 148 (9.5) | 371 (11.7) |
|  | **Japan/China** | 9656 (28.5) | 3435 (24.4) | 4820 (31.9) | 285 (18.3) | 1116 (35.3) |
|  | **Europe^#1^** | 15766 (46.5) | 7063 (50.2) | 6384 (42.2) | 944 (60.7) | 1375 (43.5) |
|  | **Others^#2^** | 3522 (10.4) | 1440 (10.2) | 1603 (10.6) | 177 (11.4) | 302 (9.5) |
| **Phase** |  |  |  |  |  |  |
|  | **Ⅳ** | 9859 (29.1) | 4250 (30.2) | 4425 (29.2) | 445 (28.6) | 739 (23.4) |
|  | **Ⅴ** | 12125 (35.8) | 5006 (35.6) | 5620 (37.1) | 438 (28.2) | 1061 (33.5) |
|  | **Ⅵ** | 6088 (18.0) | 2286 (16.3) | 2802 (18.5) | 291 (18.7) | 709 (22.4) |
|  | **Ⅶ** | 5834 (17.2) | 2515 (17.9) | 2284 (15.1) | 380 (24.5) | 655 (20.7) |
| **HD vintage (years)** |  | 2.06 [0.42, 5.33] | 1.25 [0.31, 3.83] | 2.09 [0.44, 5.17] | 4.71 [2.05, 8.99] | 6.11 [2.91, 11.24] |
| **BMI (kg/m^2^)** |  | 24.37 [21.26, 28.47] | 24.35 [21.28, 28.45] | 24.31 [21.22, 28.34] | 25.13 [21.73, 29.41] | 24.30 [21.21, 28.68] |
| **Sp Kt/V** |  | 1.45 [1.24, 1.67] | 1.43 [1.21, 1.66] | 1.43 [1.24, 1.66] | 1.52 [1.32, 1.73] | 1.55 [1.37, 1.76] |
| ***Comorbidities*** |  |  |  |  |  |  |
|  | **Diabetes** | 14460 (42.6) | 6310 (44.9) | 6540 (43.2) | 544 (35.0) | 1066 (33.7) |
|  | **Hypertension** | 29281 (86.4) | 12138 (86.3) | 13063 (86.3) | 1346 (86.6) | 2734 (86.4) |
|  | **Coronary heart disease** | 11470 (33.8) | 5018 (35.7) | 4961 (32.8) | 501 (32.2) | 990 (31.3) |
|  | **Congestive heart failure** | 7251 (21.4) | 3281 (23.3) | 3097 (20.5) | 309 (19.9) | 564 (17.8) |
|  | **Other Cardiovascular disease** | 9263 (27.3) | 3912 (27.8) | 3989 (26.4) | 461 (29.7) | 901 (28.5) |
|  | **Cerebrovascular disease** | 5092 (15.0) | 2225 (15.8) | 2233 (14.8) | 214 (13.8) | 420 (13.3) |
|  | **Peripheral vascular disease** | 8126 (24.0) | 3618 (25.7) | 3374 (22.3) | 381 (24.5) | 753 (23.8) |
|  | **Cancer** | 4708 (13.9) | 2127 (15.1) | 1985 (13.1) | 208 (13.4) | 388 (12.3) |
|  | **Neurologic disease** | 3385 (10.0) | 1613 (11.5) | 1368 (9.0) | 150 (9.7) | 254 (8.0) |
|  | **Lung disease** | 3924 (11.6) | 1873 (13.3) | 1589 (10.5) | 175 (11.3) | 287 (9.1) |
|  | **History of hip/vertebral fractures** | 1644 (4.8) | 714 (5.1) | 705 (4.7) | 85 (5.5) | 140 (4.4) |
| ***Laboratory*** |  |  |  |  |  |  |
|  | **Hemoglobin (g/dL)** | 11.00 [10.10, 11.90] | 10.90 [9.90, 11.80] | 11.00 [10.10, 11.90] | 11.30 [10.40, 12.20] | 11.20 [10.40, 12.00] |
|  | **BUN (mg/dL)** | 56.00 [44.00, 68.60] | 55.00 [42.84, 68.40] | 56.00 [44.00, 68.04] | 59.27 [47.60, 72.00] | 58.00 [47.13, 70.00] |
|  | **Cre (mg/dL)** | 8.03 [6.13, 10.19] | 7.49 [5.66, 9.61] | 8.10 [6.30, 10.20] | 9.05 [7.24, 11.02] | 9.48 [7.62, 11.57] |
|  | **Ca (mg/dL)** | 8.88 [8.40, 9.36] | 8.80 [8.40, 9.30] | 8.90 [8.44, 9.40] | 8.80 [8.30, 9.32] | 8.88 [8.40, 9.40] |
|  | **Phosphorus (mg/dL)** | 4.96 [4.06, 6.00] | 4.80 [3.93, 5.92] | 5.00 [4.10, 6.00] | 5.11 [4.10, 6.50] | 5.20 [4.30, 6.10] |
|  | **i-PTH (pg/mL)** | 233.70 [126.00, 418.00] | 197.00 [107.00, 337.32] | 250.00 [137.00, 435.00] | 380.00 [203.05, 738.00] | 294.50 [154.00, 568.00] |
| ***Medications*** |  |  |  |  |  |  |
|  | **ESA use** | 28461 (85.0) | 11670 (84.7) | 12961 (86.3) | 1232 (80.3) | 2598 (82.9) |
|  | **Phosphate-binder use** | 24228 (72.1) | 8826 (63.6) | 11354 (75.5) | 1278 (83.1) | 2770 (88.1) |

Values are median [interquartile range], or number (proportion).

Abbreviations: BMI, body mass index; BUN, blood urea nitrogen; Ca, albumin-corrected serum calcium; Cre, serum creatinine; ESA, erythropoiesis-stimulating agent; HD vintage, years since initiation of maintenance haemodialysis; i-PTH, intact parathyroid hormone; spKt/V, single-pool Kt/V (dialysis adequacy index); SMD, standardised mean difference; VDRA, vitamin D receptor activator.

#1 Europe: Belgium, France, Germany, Italy, Spain, Sweden and UK

#2 Others: Australia-New Zealand, GCC, Russia and Turkey

**Supplemental Table S6.** **Number of patients in each treatment category over time**

| **Month** | **Total (n)** | **Missing (n)** | **Combination (n)** | **Combination (%)** | **VDRA (n)** | **VDRA (%)** | **Calcimimetics (n)** | **Calcimimetics (%)** | **Neither (n)** | **Neither (%)** |
| --- | --- | --- | --- | --- | --- | --- | --- | --- | --- | --- |
| **0** | 117452 | 0 | 14501 | 12.30% | 57265 | 48.80% | 4234 | 3.60% | 41452 | 35.30% |
| **1** | 105192 | 695 | 14303 | 13.70% | 52355 | 50.10% | 3832 | 3.70% | 33953 | 32.50% |
| **2** | 100163 | 788 | 14483 | 14.60% | 50166 | 50.50% | 3726 | 3.80% | 30946 | 31.20% |
| **3** | 95634 | 749 | 14567 | 15.40% | 48165 | 50.80% | 3656 | 3.90% | 28437 | 30.00% |
| **4** | 91872 | 721 | 14656 | 16.10% | 46336 | 50.90% | 3579 | 3.90% | 26499 | 29.10% |
| **5** | 84450 | 899 | 13951 | 16.70% | 42701 | 51.10% | 3324 | 4.00% | 23510 | 28.20% |
| **6** | 81267 | 852 | 13833 | 17.20% | 41269 | 51.40% | 3230 | 4.00% | 22008 | 27.40% |
| **7** | 77205 | 739 | 13556 | 17.70% | 39402 | 51.60% | 3129 | 4.10% | 20308 | 26.60% |
| **8** | 74705 | 718 | 13383 | 18.10% | 38087 | 51.50% | 3111 | 4.20% | 19305 | 26.10% |
| **9** | 71428 | 718 | 13047 | 18.50% | 36344 | 51.50% | 3028 | 4.30% | 18216 | 25.80% |
| **10** | 69149 | 771 | 12917 | 18.90% | 35226 | 51.60% | 2974 | 4.40% | 17183 | 25.20% |
| **11** | 66865 | 661 | 12856 | 19.40% | 34207 | 51.70% | 2884 | 4.40% | 16181 | 24.50% |
| **12** | 64602 | 630 | 12673 | 19.80% | 33133 | 51.90% | 2816 | 4.40% | 15255 | 23.90% |
| **13** | 55484 | 717 | 11468 | 21.00% | 28629 | 52.30% | 2384 | 4.40% | 12230 | 22.40% |
| **14** | 53673 | 753 | 11290 | 21.40% | 27701 | 52.40% | 2329 | 4.40% | 11548 | 21.80% |
| **15** | 51863 | 663 | 11190 | 21.90% | 26640 | 52.10% | 2291 | 4.50% | 11036 | 21.60% |
| **16** | 50179 | 648 | 11052 | 22.30% | 25760 | 52.10% | 2201 | 4.40% | 10467 | 21.20% |
| **17** | 45956 | 623 | 10448 | 23.10% | 23400 | 51.70% | 2047 | 4.50% | 9398 | 20.70% |
| **18** | 44428 | 621 | 10241 | 23.40% | 22551 | 51.50% | 1999 | 4.60% | 8974 | 20.50% |
| **19** | 41814 | 603 | 9820 | 23.90% | 21263 | 51.60% | 1904 | 4.60% | 8186 | 19.90% |
| **20** | 40429 | 584 | 9662 | 24.30% | 20422 | 51.30% | 1881 | 4.70% | 7831 | 19.70% |
| **21** | 37444 | 355 | 9241 | 24.90% | 18765 | 50.60% | 1806 | 4.90% | 7237 | 19.50% |
| **22** | 36336 | 350 | 9105 | 25.30% | 18097 | 50.30% | 1786 | 5.00% | 6960 | 19.40% |
| **23** | 35287 | 297 | 8982 | 25.70% | 17538 | 50.20% | 1749 | 5.00% | 6679 | 19.10% |
| **24** | 34161 | 274 | 8790 | 26.00% | 16907 | 50.00% | 1763 | 5.20% | 6384 | 18.90% |

**Supplemental Table S7. Drug continuation rates by follow-up period**

| **Baseline pattern** | **Patients*** **(n)** | **Continued patients (n)** | **Continuity rate (%)** | **Period** |
| --- | --- | --- | --- | --- |
| Neither | 27088 | 18770 | 69.3 | 6 |
| VDRA only | 41731 | 33169 | 79.5 | 6 |
| Calcimimetics only | 3093 | 1539 | 49.8 | 6 |
| Combination | 11416 | 8996 | 78.8 | 6 |
| Neither | 21758 | 11701 | 53.8 | 12 |
| VDRA only | 34694 | 23330 | 67.2 | 12 |
| Calcimimetics only | 2563 | 852 | 33.2 | 12 |
| Combination | 9661 | 6231 | 64.5 | 12 |
| VDRA only | 26526 | 15809 | 59.6 | 18 |
| Neither | 15601 | 6773 | 43.4 | 18 |
| Calcimimetics only | 1927 | 451 | 23.4 | 18 |
| Combination | 7775 | 4396 | 56.5 | 18 |
| VDRA only | 21273 | 11283 | 53 | 24 |
| Neither | 12097 | 4452 | 36.8 | 24 |
| Calcimimetics only | 1609 | 285 | 17.7 | 24 |
| Combination | 6490 | 3247 | 50 | 24 |

Continuation rate was defined as the proportion of patients who maintained their baseline drug pattern without any changes throughout the specified period.

*Patients with <80% of the respective follow-up period were excluded from each time-point analysis to ensure reliable estimation of continuation rates.

**Supplemental Table S8.** Distribution of stabilized time-point and cumulative weights by treatment category

| **Treatment group** | **N** | **Mean (SD)** | **Median** | **IQR** | **1st–99th pct** | **Min–Max** | **% Truncated** |
| --- | --- | --- | --- | --- | --- | --- | --- |
| **All-cause mortality cohort** | | | | | | | |
| *Time-point weight (w_t) — truncated at 2nd/98th percentile* | | | | | | | |
| Neither | 469,774 | 0.995 (0.056) | 0.995 | 0.973–1.021 | 0.735–1.116 | 0.027–11.294 | 4.69 |
| VDRA only | 957,618 | 0.996 (0.040) | 0.996 | 0.990–1.005 | 0.735–1.116 | 0.061–41.915 | 2.75 |
| Calcimimetic only | 82,794 | 0.984 (0.088) | 0.995 | 0.954–1.036 | 0.735–1.116 | 0.012–9.715 | 12.62 |
| Both | 385,281 | 0.993 (0.052) | 0.995 | 0.985–1.008 | 0.735–1.116 | 0.094–22.488 | 4.42 |
| *Cumulative weight (SW_t) — truncated at 1st/99th percentile* | | | | | | | |
| Neither | 469,774 | 0.958 (0.237) | 0.960 | 0.833–1.053 | 0.369–1.774 | 0.044–56.150 | 2.44 |
| VDRA only | 957,618 | 0.954 (0.183) | 0.974 | 0.882–1.021 | 0.441–1.575 | 0.053–9.835 | 0.98 |
| Calcimimetic only | 82,794 | 0.901 (0.331) | 0.888 | 0.674–1.075 | 0.355–1.774 | 0.011–48.543 | 7.99 |
| Both | 385,281 | 0.910 (0.247) | 0.935 | 0.752–1.028 | 0.355–1.772 | 0.033–12.687 | 2.73 |
| **CVD mortality cohort** | | | | | | | |
| *Time-point weight (w_t) — truncated at 2nd/98th percentile* | | | | | | | |
| Neither | 234,485 | 0.998 (0.029) | 0.998 | 0.984–1.013 | 0.874–1.069 | 0.049–8.688 | 4.41 |
| VDRA only | 430,484 | 0.999 (0.020) | 0.998 | 0.993–1.004 | 0.874–1.069 | 0.091–13.735 | 2.79 |
| Calcimimetic only | 39,029 | 0.994 (0.044) | 1.001 | 0.979–1.018 | 0.874–1.069 | 0.022–5.949 | 11.40 |
| Both | 169,002 | 0.997 (0.027) | 0.996 | 0.989–1.006 | 0.874–1.069 | 0.132–13.619 | 4.81 |
| *Cumulative weight (SW_t) — truncated at 1st/99th percentile* | | | | | | | |
| Neither | 234,485 | 0.987 (0.159) | 0.983 | 0.903–1.050 | 0.611–1.535 | 0.109–9.205 | 2.99 |
| VDRA only | 430,484 | 0.982 (0.114) | 0.986 | 0.932–1.022 | 0.682–1.388 | 0.187–7.059 | 0.81 |
| Calcimimetic only | 39,029 | 0.976 (0.200) | 0.971 | 0.853–1.070 | 0.611–1.535 | 0.084–8.512 | 6.89 |
| Both | 169,002 | 0.962 (0.160) | 0.963 | 0.867–1.029 | 0.611–1.535 | 0.201–7.728 | 2.52 |
| **Fracture cohort** | | | | | | | |
| *Time-point weight (w_t) — truncated at 2nd/98th percentile* | | | | | | | |
| Neither | 186,633 | 0.999 (0.022) | 0.998 | 0.988–1.010 | 0.898–1.056 | 0.109–5.051 | 3.64 |
| VDRA only | 256,607 | 0.999 (0.017) | 0.999 | 0.995–1.004 | 0.898–1.056 | 0.144–5.569 | 2.96 |
| Calcimimetic only | 32,281 | 0.996 (0.032) | 0.999 | 0.984–1.014 | 0.898–1.056 | 0.139–5.736 | 9.37 |
| Both | 84,678 | 0.997 (0.024) | 0.997 | 0.990–1.006 | 0.898–1.056 | 0.171–5.921 | 5.92 |
| *Cumulative weight (SW_t) — truncated at 1st/99th percentile* | | | | | | | |
| Neither | 186,633 | 0.994 (0.129) | 0.990 | 0.927–1.048 | 0.677–1.442 | 0.288–3.519 | 2.42 |
| VDRA only | 256,607 | 0.989 (0.091) | 0.991 | 0.948–1.022 | 0.751–1.303 | 0.467–3.221 | 0.60 |
| Calcimimetic only | 32,281 | 0.976 (0.158) | 0.974 | 0.881–1.049 | 0.677–1.442 | 0.158–3.242 | 5.85 |
| Both | 84,678 | 0.972 (0.142) | 0.969 | 0.889–1.028 | 0.677–1.442 | 0.336–4.773 | 3.85 |

Values are averaged across 20 multiply imputed datasets. Time-point weights (w_t) were truncated at the 2nd and 98th percentiles; cumulative weights (SW_t) were truncated at the 1st and 99th percentiles. The percentage truncated represents the proportion of person-month observations falling outside the respective truncation bounds. Min–Max values reflect the pre-truncation weight distributions.

Abbreviations: SW_t, cumulative stabilized weight; w_t, time-point stabilized weight.

**Supplemental Table S9.** Indication-restricted sensitivity analysis of all-cause mortality among patients with baseline i-PTH ≥300 pg/mL

| **Exposure** | **Number of patients^a^, n** | **Number of events^a^, n** | **Follow-up mean (SD)^a^, months** | **Event rate^a^, 100 patient-years (95%CI)** | **Hazard ratio (95% CI)** | | | |
| --- | --- | --- | --- | --- | --- | --- | --- | --- |
|  |  |  |  |  | **Crude^b^** | **p-value** | **Weighted^c^** | **p-value** |
|  |  |  |  |  |  |  |  |  |
| **Neither use** | 20,379 | 1,887 | 6.1 (7.0) | 18.21 (17.40–19.05) | Ref |  | Ref |  |
| **VDRA only** | 40,652 | 5,284 | 11.4 (10.5) | 13.68 (13.31–14.05) | 0.69 (0.65–0.73) | <0.001 | 0.75 (0.70–0.80) | <0.001 |
| **Calcimimetics only** | 8,231 | 639 | 6.0 (6.8) | 15.42 (14.25–16.66) | 0.78 (0.71–0.86) | <0.001 | 0.81 (0.73–0.90) | <0.001 |
| **Combination therapy** | 19,366 | 2,444 | 12.9 (11.3) | 11.72 (11.26–12.20) | 0.56 (0.53–0.60) | <0.001 | 0.62 (0.58–0.67) | <0.001 |
|  |  |  |  |  |  |  |  |  |

Restriction was defined using baseline i-PTH. The unweighted eligible cohort included 57,425 patients (48.9% of the all-cause mortality cohort). Numbers of patients, events, follow-up periods, and event rates were calculated in the weighted population.

a Number of patients, events, follow up periods, event rate were calculated in the weighted population

b Crude models were stratified by Country region and used robust variance estimation with clustering at the facility level.

c Adjustments for confounders: age, sex, race, country, DOPPS phase, hemodialysis vintage (years), body mass index (BMI), single-pool Kt/v, comorbidi-ties (diabetes, hypertension, coronary heart disease, congestive heart failure, cerebrovascular disease, other cardiovascular disease, peripheral vascular dis-ease, cancer, neurologic disease, lung disease, and hip/vertebral fractures), laboratory data (hemoglobin, blood urea nitrogen (BUN), creatinine, albumin-corrected calcium, phosphate and i-PTH ) , erythropoiesis-stimulating agent (ESA) use, and phosphate binder use. The weighted Cox models were strati-fied by country region and used robust variance estimation with clustering at the facility level.

Abbreviations: CI, confidence interval; HR, hazard ratio; mo, month; n, number; PY, patient-years; SD, standard deviation; VDRA, vitamin D receptor activator; yr, year; ref, reference group.

**Supplemental Table S10.** Complementary Fine–Gray subdistribution hazard analysis for CVD mortality using baseline treatment category

| Comparison | Subdistribution hazard ratio (95% CI) | p-value |
| --- | --- | --- |
| VDRA only vs. Neither | 0.94 (0.87–1.03) | 0.195 |
| Calcimimetics only vs. Neither | 0.99 (0.81–1.22) | 0.927 |
| Combination therapy vs. Neither | 0.94 (0.83–1.07) | 0.342 |

Values are pooled across 20 multiply imputed datasets using Rubin’s rules. Fine–Gray subdistribution hazard models used baseline treatment category, with non-CVD death treated as the competing event, and were adjusted for age, sex, dialysis vintage, country/region, and DOPPS phase. Reference group: neither use.

**Abbreviations**: CVD, cardiovascular disease; SHR, subdistribution hazard ratio; VDRA, vitamin D receptor activator.
